# Identification and Validation of EMT-Related lncRNA Prognostic Signature for Colorectal Cancer

**DOI:** 10.3389/fgene.2021.723802

**Published:** 2021-09-22

**Authors:** Danfeng Li, Xiaosheng Lin, Binlie Chen, Zhiyan Ma, Yongming Zeng, Huaiming Wang

**Affiliations:** ^1^ Department of Gastrointestinal Surgery, The First Affiliated Hospital of Shantou University Medical College, Shantou, China; ^2^ Medical College, Shantou University, Shantou, China

**Keywords:** EMT-related lncRNA, nomogram, colorectal cancer, prognostic, signature

## Abstract

**Background:** This study aimed to explore the biological functions and prognostic role of Epithelial-mesenchymal transition (Epithelial-mesenchymal transition)-related lncRNAs in colorectal cancer (CRC).

**Methods:** The Cancer Genome Atlas database was applied to retrieve gene expression data and clinical information. An EMT-related lncRNA risk signature was constructed relying on univariate Cox regression, Least Absolute Shrinkage and Selector Operation (LASSO) and multivariate Cox regression analysis of the EMT-related lncRNA expression data and clinical information. Then, an individualized prognostic prediction model based on the nomogram was developed and the predictive accuracy and discriminative ability of the nomogram were determined by the receiver operating characteristic curve and calibration curve. Finally, a series of analyses, such as functional analysis and unsupervised cluster analysis, were conducted to explore the influence of independent lncRNAs on CRC.

**Results:** A total of 581 patients were enrolled and an eleven-EMT-related lncRNA risk signature was identified relying on the comprehensive analysis of the EMT-related lncRNA expression data and clinical information in the training cohort. Then, risk scores were calculated to divide patients into high and low-risk groups, and the Kaplan-Meier curve analysis showed that low-risk patients tended to have better overall survival (OS). Multivariate Cox regression analysis indicated that the EMT-related lncRNA signature was significantly associated with prognosis. The results were subsequently confirmed in the validation dataset. Then, we constructed and validated a predictive nomogram for overall survival based on the clinical factors and risk signature. Functional characterization confirmed this signature could predict immune-related phenotype and was associated with immune cell infiltration (i.e., macrophages M0, M1, Tregs, CD4 memory resting cells, and neutrophils), tumor mutation burden (TMB).

**Conclusions:** Our study highlighted the value of the 11-EMT-lncRNA signature as a predictor of prognosis and immunotherapeutic response in CRC.

## Introduction

Colorectal cancer (CRC) is the third most common malignancy with the second-highest cancer-related mortality worldwide. The number of cases is expected to rise by 60% in the year 2030 worldwide. Despite the development of CRC therapies such as surgery, radiotherapy, chemotherapy, targeted therapy, immunotherapy, the 5-years survival rate of late stages CRC still less than 20% (Siegel et al., 2017). Increasing data underline that the tumor microenvironment (TME) contributes a vital role in CRC progression, as well as in the response to therapy. In most types of cancer, the infiltration of CD8 T cells and tumor-infiltrating lymphocytes (TIL) in tumor beds is a biomarker for a good prognosis (Ma et al., 2019). Similarly, the presence of CD8 T cells in the tumor bed and infiltrating margins is strongly associated with prognosis in CRC (Zhang et al., 2018).

Based on the mutation pattern and the ratio of MSI markers, CRC tumors can be divided into the dMMR group and pMMR group. In recent years, studies have shown that dMMR-MSI-H CRC tumors have a high tumor mutation burden and can present new antigens on major histocompatibility complex (MHC) class I molecules, which makes them more sensitive to T cell activation therapy, while the pMMR-MSI-L CRC tumor has a low tumor mutation burden, with a low immune response rate (Ledys et al., 2018). Therefore, in 2017, the Food and Drug Administration (FDA) approved PD-1 drugs for dMMR-MSI-H mCRC patients. Unfortunately, only about 15% of CRC patients with the dMMR-MSI-H phenotype, and among all mCRC patients, the dMMR-MSI-H phenotype only accounts for about 5%. Moreover, not all CRC cases with the dMMR-MSI-H phenotype respond well to immunotherapies (Fabrizio et al., 2018). A series of studies showed that the effective rate of immunotherapy in CRC with dMMR-MSI-H phenotype is only 40%, while in pMMR CRC patients, the effective rate of immunotherapy is very low, and recent biological advances suggest that combination therapy can reverse this resistance (Ghiringhelli and Fumet, 2019). Furthermore, several experimental data have shown that tumor intrinsic factors may also modulate responses to immunotherapy, such as genes participating in cell adhesion, extracellular matrix remodeling, angiogenesis, wound healing, and mesenchymal transformation (Hugo et al., 2016). As such, it is urgent to look for biomarkers or more effective strategies based on tumor gene-expression profiling to treat patients with various subsets of advanced CRC.

Epithelial-mesenchymal transition (EMT) is a process in which epithelial cells lose connection and polarity, and acquire plasticity, migration, invasion ability, stem cell-like characteristics, and resistance to apoptosis. It has been proved that EMT is an important way to promote tumor cell metastasis. Accumulating preclinical researches have confirmed that the level of EMT contributes to the level of immunosuppression, with more mesenchymal tumors being more resistant to immunotherapy, and tumor immunosuppression and immune evasion could be reversed by the EMT progress. (Terry et al., 2017; Dongre and Weinberg, 2019).

Moreover, emerging studies revealed that EMT is regulated by a complex regulatory network, including the typical regulation of EMT transcription factors (EMT-TFs), noncoding RNAs, epigenetic modification, post-translational regulation, and alternative splicing factors (Chaffer et al., 2016; Diepenbruck and Christofori, 2016; Nieto et al., 2016). Thus, EMT-related lncRNAs and genes may be a promising target for future therapeutic interventions.

Currently, accumulating shreds of evidence indicated that lncRNAs play a vital role in the progression of tumors and can be used as robust predictors of the prognosis for cancer patients. It has been well known that lncRNAs involved EMT progression. For instance, in our previous study, we identified that linc00662 was significantly increased in CRC cells and tissues, and significantly stimulating EMT progression and inducing tumor growth both *in vivo* and *in vitro* (Wang et al., 2020a). Moreover, other research revealed that decreasing the expression of linc01133 can inhibit EMT and metastasis in CRC cells (Kong et al., 2016). Nevertheless, a single lncRNA may only explain its partial effect on tumors, so it is very important to comprehensively analyze the expression profile of EMT-related lncRNAs, as well as their different pathological features and prognostic value in CRC, which may lead to a deeper understanding of the effect of EMT-related lncRNAs on tumors and to propose newer treatment strategies.

In the current research, we analyzed the RNAseq data and corresponding clinical information retrieved from the TCGA (N = 581) database to comprehensively explore the prognostic role of EMT-related lncRNA, and an 11-lncRNA signature was constituted and validated in the training and test cohorts. Furthermore, we then characterized the underlying molecular and immune profile of EMT-related lncRNAs signature in CRC. Consequently, we found that this signature could identify different immune infiltration states, TIDE prediction score, and MSI status of each patient, which explains it was a promising prognostic biomarker for CRC patients receiving immunotherapy.

## Methods

### Data Acquisition

The RNA-seq reads count and clinical information were obtained from the TCGA database (https://portal.gdc.cancer.gov/). Samples with a survival time≥of 30 days were selected to ensure higher quality analysis. Subsequently, 581 patients with CRC from the TCGA were included for further analysis. Then, we retrieved somatic mutation profiles of all tumor samples in the TCGA database.

### Identification of Epithelial-Mesenchymal Transition-Related lncRNAs

200 EMT-related genes were downloaded from the Molecular Signature database v7.1 (MSigDB) (http://www.broad.mit.edu/gsea/msigdb/). To identify EMT-related lncRNAs, firstly, all lncRNAs expression data were extracted from the TCGA database relying on the GENCODE project (http://www.gencodegenes.org). Then, Pearson correlation analysis between EMT-related genes and all lncRNA expression data in samples was performed to identify the EMT-related lncRNA based on |Cor pearson| > 0.6 and *p*-value < 0.01.

### Development and Validation of the Prognostic Signature

CRC patients were randomly divided into training and test cohorts with a 6:4 ratio. In the training cohort, univariate Cox regression analysis was carried out to explore the relationship between each EMT-related lncRNA expression and overall survival (OS). Then, these lncRNAs were further analyzed by utilizing LASSO penalized Cox proportional hazards regression to identify the best risk model in the R package “glmnet”. Using the following formula: risk score=(β1*G1+β2*G2+β3*G3+⋯+βn*Gn) to calculate the risk score for each patient, where β is the coefficient of each lncRNA, G represents each lncRNA expression value, and n denotes the number of lncRNAs. Patients were classified into two risk groups depending on the median risk score. Moreover, the survival curve was adopted using the Kaplan-Meier method in the R survminer package, where the differences between the two risk groups were calculated by the log-rank test. Meanwhile, a time-dependent receiver operating characteristic (ROC) curve was determined using R ‘survivalROC’ package, of which the area under the curve (AUC) was calculated to assess the accuracy of the prognostic risk signature. To further verify the predictive performance of the prognostic signature, the risk scores were also calculated in the testing cohort utilizing the same prognostic formula, and the Kaplan–Meier survival curve and ROC curve were conducted with a cutoff value of the median risk score.

#### Independence of the Epithelial-Mesenchymal Transition-Related lncRNA Signature

Univariate Cox regression analysis and multivariate Cox regression analysis were used to identify independence by exploiting the lncRNA characteristics of OS and corresponding clinical information. *p* < 0.05 was considered as statistically significant.

### Nomogram Construction and Validation

The ‘rms’ R package was used to establish the nomogram based on all independent prognostic factors (https://cran.r-project.org/web/packages/rms/index.html). Then, Calibration plot curve analysis was applied to evaluate the discrimination and the calibration of the nomogram.

### Gene Set Enrichment Analysis Enrichment Analysis

In the signaling pathway analysis, differential expression analysis was first performed on all genes to analyze the samples with the high and low-risk score using the ‘DESeq2’ package of R. Enrichment analysis to determine the signaling pathways in which the differentially expressed genes are involved was then carried out by using the gene set enrichment analysis (GSEA) method based on the HALLMARK gene sets with the ‘clusterProfiler’ package of R. When *p* < 0.05 and FDR <0.05, the path ways were considered as statistically significant.

### Gene Mutation Analysis

In the gene mutation analysis, information on genetic alterations was obtained from the cBioPortal database, and the quantity and quality of gene mutations in two risk subgroups were analyzed by utilizing the ‘Maftools’ package of R. Then, we calculated the TMB of each patient and described the difference of TMB in two risk subgroups.

### Tumor Microenvironment Analysis

To evaluate the tumor microenvironment in CRC, we identified the infiltration levels of 22 immune cells using the CIBERSORT algorithm based on the expression level of all genes. First, we uploaded the expression data of all genes to the CIBERSORTx web portal. Next, the algorithm was run using the LM22 signature for 1,000 permutations. The CRC samples with an output *p*-value < 0.05 were selected for further analysis. Moreover, the immune core and the stromal score were calculated using the “estimate” R package. Single sample GSEA (ssGSEA) analysis was then performed with the ‘GSVA’ package of R, to estimate the abundance of 28 immune infiltrate cells. Additionally, TIMER 2.0 (Tumor Immune Estimation Resource) database was used to explore the correlation of mutation genes and immune infiltration level in CRC.

### Immunotherapeutic Sensitivity With Prognostic Signature

To further validate the predictive performance of the given prognostic signature for the ICIs response, the Tumor Immune Dysfunction and Exclusion (TIDE) algorithm was assigned to assess the immunogenicity and immunotherapeutic sensitivity of CRC patients. The results were measured by the TIDE score, which was calculated online (http://tide.dfci.harvard.edu/). According to the default settings, a patient with a TIDE value < 0 was defined as a responder (positive sensitivity to immunotherapy), whereas a patient with a TIDE value > 0 was defined as a non-responder (negative sensitivity to immunotherapy).

### Statistical Analysis

R software (R version: 3.6.3) was used to perform all data statistical analyses. Wilcoxon test (Mann-Whitney test) was applied to analyze continuous variables, whereas the Fisher’s exact test or chi-square test was used to analyze the categorical data. The survival difference was calculated with the K-M analysis methods and the log-rank test. For all statistical analyses, *p*-value less than 0.05 indicated statistical significance.

## Results

### Identified Epithelial-Mesenchymal Transition-Related lncRNA in CRC

To explore EMT-Related genes in CRC, we initially retrieved the data from the MSigDB database, with the hallmark gene sets name: HALLMARK_EPITHELIAL_MESENCHYMAL_TRANSITION, we collected altogether 200 EMT-related genes (Supplementary Table S1). Then, we carried out correlation analysis on EMT-related genes and EMT-related lncRNAs, and the absolute Pearson coefficient >0.6 and *p*-value < 0.01 was used as the screening criteria, we identified a total of 1381 EMT-related lncRNAs (Supplementary Table S2). Finally, we merged the LncRNA expression data and clinical information for further analysis.

### Construction and Validation of the EMT-Related lncRNA Signature

**FIGURE 1:**
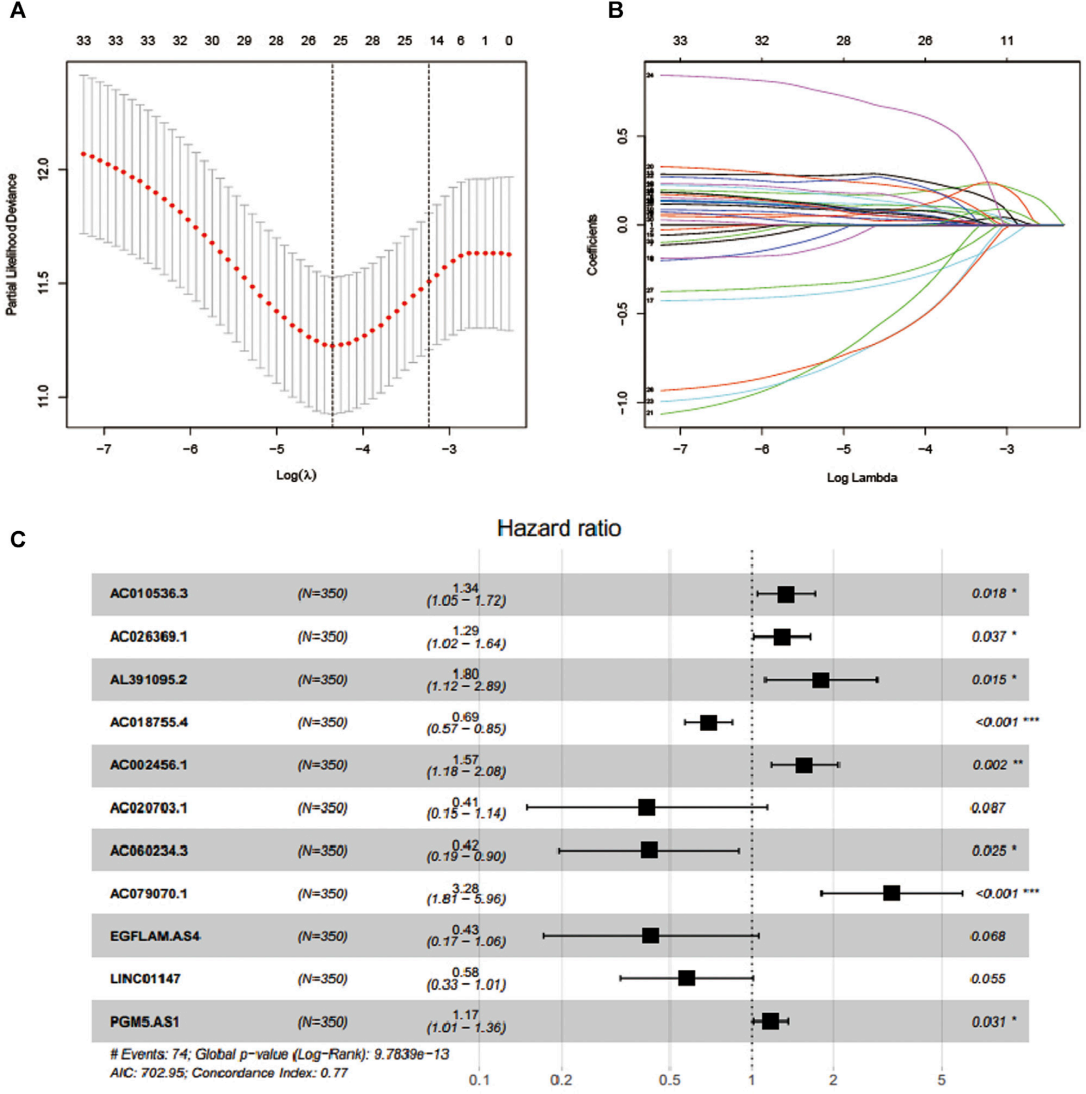
Feature selection using LASSO COX regression and multivariate Cox regression analysis. **(A)** Selection of tuning parameter (*λ*) in the LASSO regression using 10-fold cross-validation via minimum criteria. **(B)** LASSO coefficient profiles for clinical features and 25 nonzero coefficients are selected. C.11 lncRNAs were selected through multivariate Cox regression analysis.

In total, 581 eligible patients with integrated information, as well as a survival time≥of 30 days were incorporated in the TCGA-CRC dataset and randomly divided into two independent cohorts at a ratio of 6:4, and 1381 EMT-related lncRNAs were included to identify the prognostic risk model. In the univariate Cox regression analyses, 34 lncRNAs were significantly related to OS, which was considered as potential predictors. Then, a LASSO regression algorithm was applied for feature selection, when the partial likelihood binomial deviation reaches the minimum value, the most suitable tuning parameter λ for LASSO regression is 0.055 (Figure 1A), 25 variables with non-zero coefficients retained in the LASSO analysis (Figure 1B) were further used for multivariate stepwise Cox regression analysis. Then, we established an 11-lncRNA signature model through multivariate stepwise Cox regression hazards analysis (Figure 1C). The risk score of each patient in the training set and validation set is calculated according to the risk formula:

Riskscore=AC010536.3∗(0.295166381)+AC026369.1∗(0.256505491)+AL391095.2∗(0.588758986)+AC018755.4∗(-0.363854498)+AC002456.1∗(0.449864493)+AC020703.1∗(-0.887819474)+AC060234.3∗(-0.871113022)+AC079070.1∗(1.188015484)+EGFLAM.AS4∗(-0.851540675)+LINC01147∗(-0.551055647)+PGM5.AS1∗(0.160723859).

**FIGURE 2:**
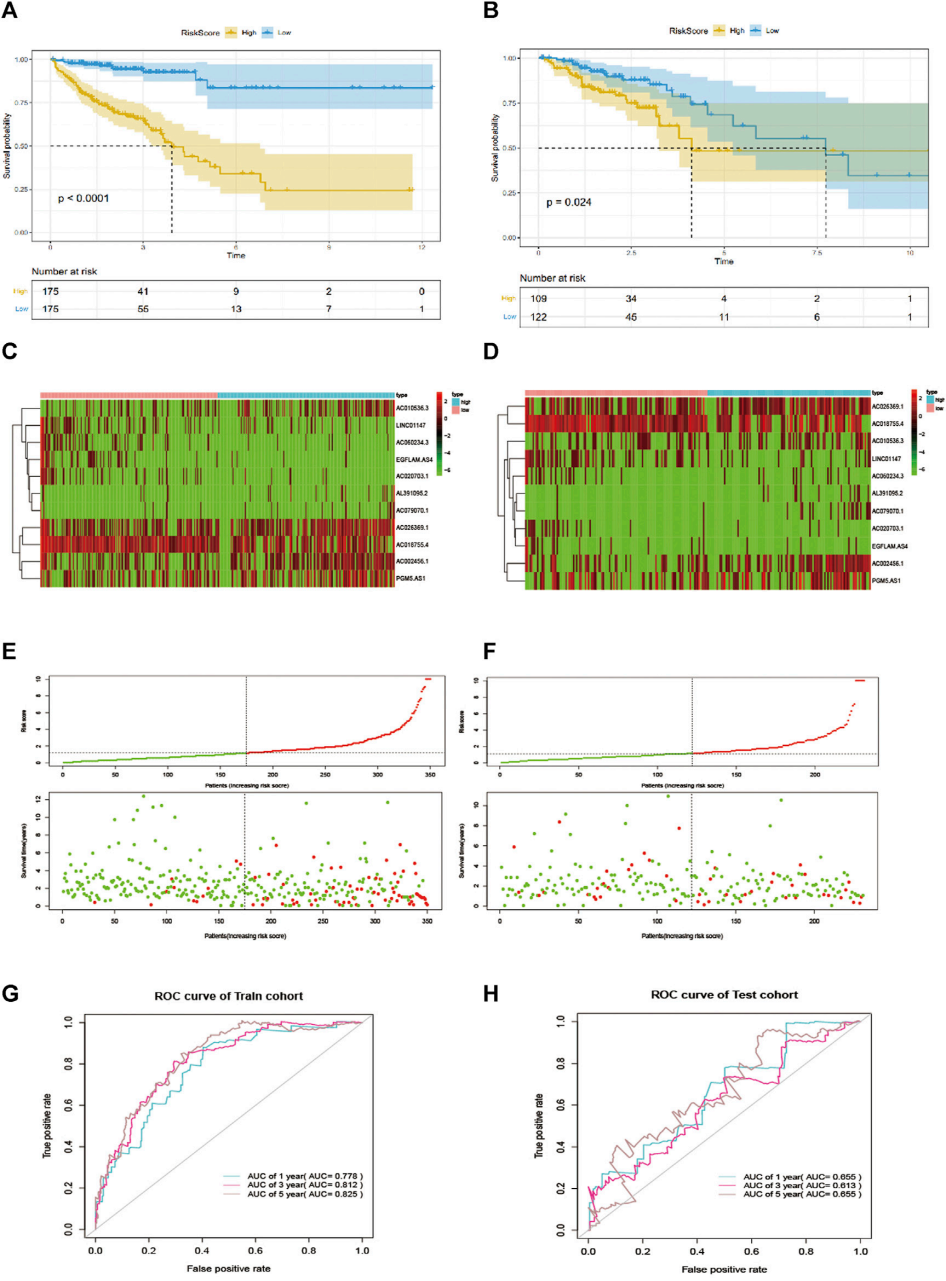
EMT-related lncRNA signature predicts OS in patients with CRC. **(A, B)** Kaplan-Meier curve to verify the predictive effect of the signature in the training and test cohort. **(C, D)** The heatmap of the expression profiles of members in the 11-lncRNA signature. **(E, F)**. Distribution of risk scores per patient in the training and test cohort. **(H, G)**: ROC curve analysis to evaluate the diagnostic efficacy of the signature.

Taking the median risk score as the cutoff value, we categorized patients into a high-risk group and low-risk group. As depicted in Figures 2A,B, our data showed that high-risk group patients had a worse OS than low-risk group patients (*p* < 0.0001 in the Training cohort and *p* = 0.024 in the testing cohort, log-rank test). Additionally, as it showed in Figure 2C, the high expression level of AC010536.3, AC026369.1, AL391095.2, AC002456.1, AC079070.1and PGM5. AS1 was reported in the high-risk group, conversely, the expression level of AC018755.4, AC020703.1, EGFLAM. AS4 and LINC01147 were higher in the low-risk group, which was consistent in the test cohort (Figure 2D). Besides, it was found the OS patients in the high-risk group have corresponded to more death cases in the training cohort and consistent in the validation cohort (Figures 2E,F). By drawing a ROC curve based on the risk model, the AUC value in the training cohort was 0.778, 0.812, 0.825, and 0.655, 0.613, 0.655 in the testing cohort in 1,3,5 year prediction, indicating a good prediction prognostic accuracy (Figures 2G,F).

**FIGURE 3:**
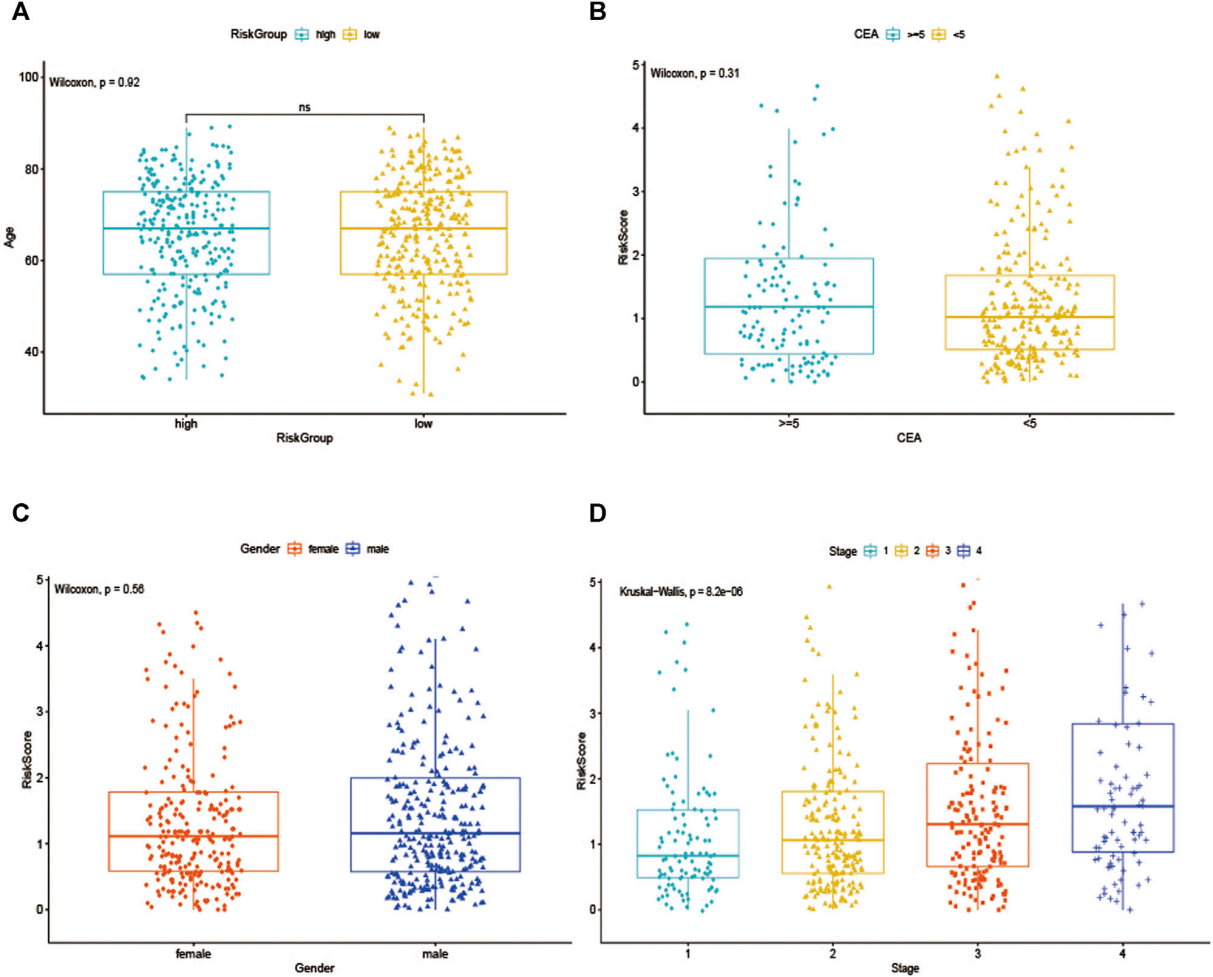
The association between EMT-related signature risk score and clinical factors, including. **(A)** Age, **(B)** CEA, **(C)** Gender, **(D)** Stage.

To further explore the prognostic value of EMT-lncRNA markers for CRC patients stratified by clinical variables, we divided patients into different groups according to age, gender, and stage, and our data showed that the risk score of CRC patients was positively associated with the stage, but no significant correlation with age, gender and plasma CEA level, considering the different stratified analysis (Figures 3A–D).

### Construction of the Nomogram and Performance

**FIGURE 4:**
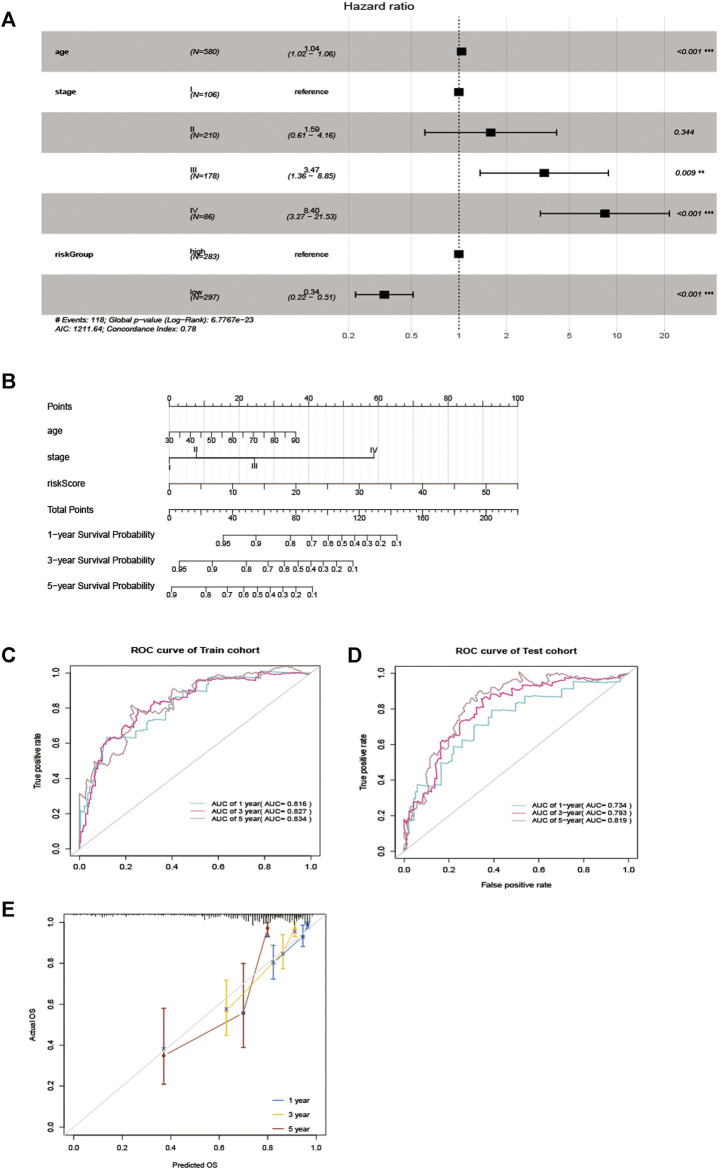
Nomogram for predicting overall survival (OS) of patients with CRC. **(A)** Multivariable analyses for each clinical feature. **(B)** Nomogram construction for the 1-, three- and 5 year OS prediction for the CRC. **(C, D)** Evaluation of the accuracy of the nomogram in 1-, three- and 5 years by using the ROC analysis. **(E)** Calibration curve for the nomogram model for predicting 1-, three- and 5 years OS.

To verify whether the EMT-related lncRNA signature can be used as an independent predictor of OS, we used univariate and multivariate Cox regression analyses. The results showed that age, stage, and the lncRNA signature can be used as independent predictors of OS (Figure 4A). Then, the EMT-related lncRNA signature, age, and stage were selected for the construction of the nomogram (Figure 4B). The AUC was 0.816,0.827,0.834 (Figure 4C) and 0.734,0.793,0.819 (Figure 4D) in the training cohort and testing cohort in predicting 1 year, 3 years, and 5 years OS in CRC, indicating good discrimination and as shown in Figure 4E, the calibration plots also present high performance in predicting 1 year, 3 years and 5 years OS in CRC. These results indicated that the nomogram has high accuracy.

### Molecular Characteristics of the Epithelial-Mesenchymal Transition-Related lncRNA Signature

**FIGURE 5:**
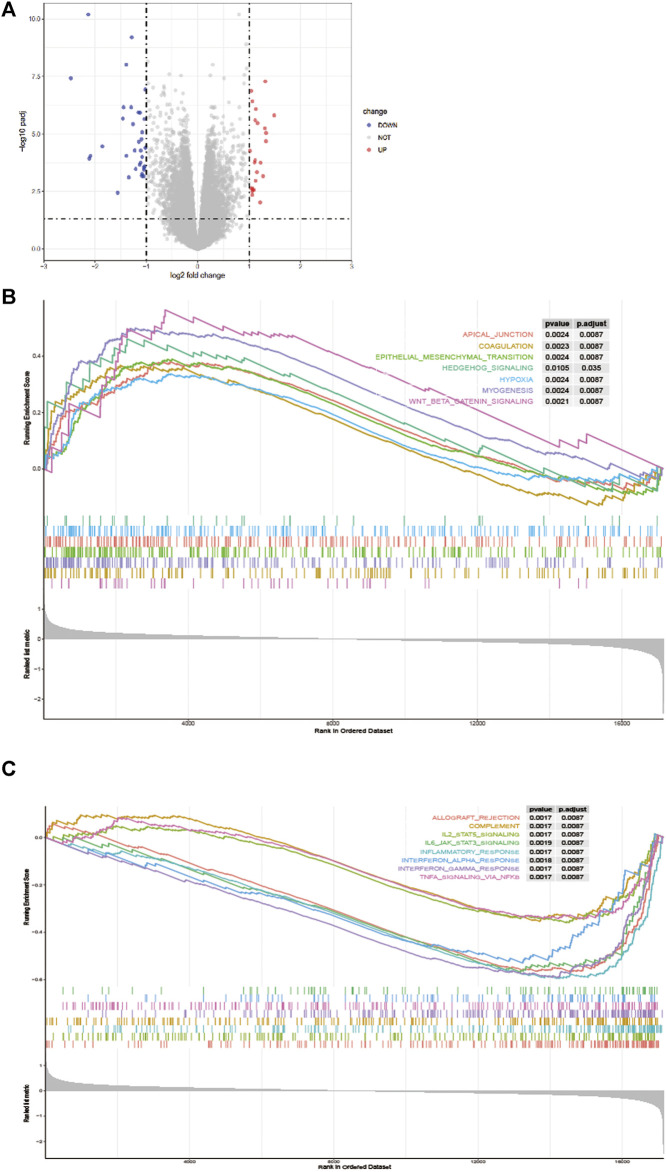
Difference analysis. **(A)** A volcano map shows different EMT-related genes of the high - and low-risk groups. Gene Set Enrichment Analysis (GSEA) for identifying the significant pathway associated with the high-risk group **(B)** and low-risk group **(C)**.

As showed in Figure 5A, in total, 58 DEGs were obtained after performing the difference analysis on the mRNA of the high - and low-risk groups, including 24 up-regulated and 34 down-regulated DEGs based on the cut-off criteria (*p* < 0.05 and |logFC|>1). Then, GSEA analysis was applied to determine the significant pathway associated with the high- and low-risk group in the training cohort, patients in the high-risk group were mainly enriched in cancer and tumor metastasis-related pathways, such as aptical_junction, coagulation, epithelial-mesenchymal transition, hedgehog_signaling, hypoxia, myog enesis and Wnt_β_catenin_signaling pathways (Figure 5B). While patients in the low-risk group were mainly enriched in immune response-related pathways, such as allograft_rejection, complement, IL2_STAT5_signaling, IL_6_JAK_STAT_3 signaling, inflammatory_response, interferon_alpha_response, interferon_gamma_response, and TNFA_signaling_via_NFKB pathways (Figure 5C).

**FIGURE 6:**
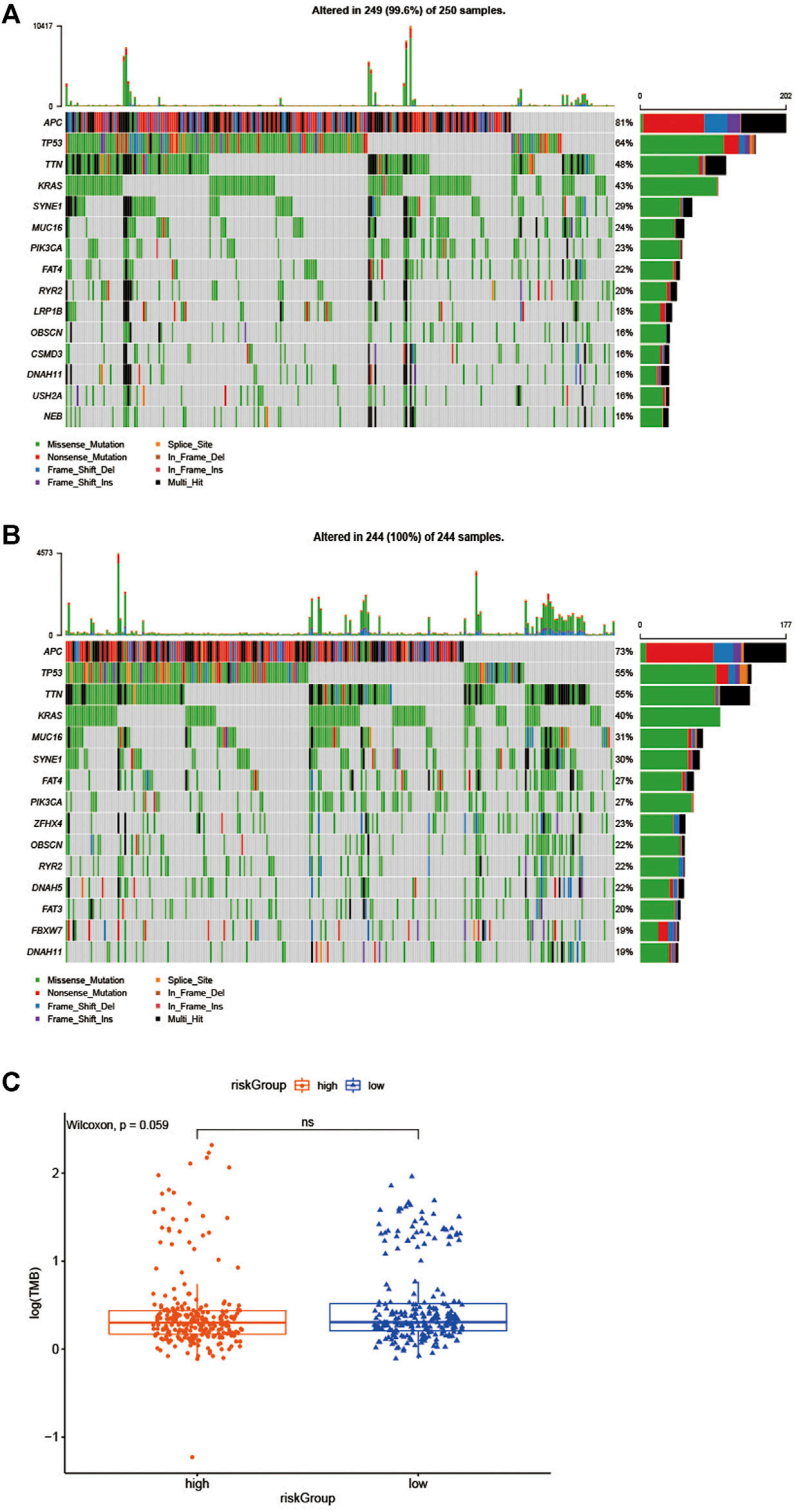
Biological insight into the high- and low-risk group. **(A, B)** significantly mutated genes in the mutated CRC samples of the high - and low-risk groups. **(C)** The proportions of TME cells in the high - and low-risk groups.

Besides, gene mutations were analyzed to gain further biological insight into the high- and low-risk group in the training cohort. The results indicated that there was no significant difference in mutation counts between the two groups, and missense_mutation was the most common type. Then, we selected the top 15 genes with the highest mutation rates in two groups (Figures 6A,B), the mutation rates of APC, TP53, TTN, KRAS, SYNE1, MUC16, PIK3CA, FAT4, RYR2, DNAH11 were both higher than 16% in the two groups. What is different is that the mutation rates of APC and TP53 were higher in the high-risk group than that in the low-risk group (81 vs 73% and 64 vs55%, which led to the decreased infiltration of CD4 + and CD8 + T lymphocytes in the high-risk group. Supplementary Figure S3), and the mutation of CSMD3, USH2A, and NEB genes were more common in the high-risk group, while the mutation of DNAH5, FAT3, and FBXW7 genes were more common in the low-risk group, Interestingly, in the TIMER 2.0 database, we found that the high mutation rate of USH2A, and NEB genes in the high-risk group resulted in decreased infiltration of CD4 + T lymphocytes and CD8 + T lymphocytes in the tumor center, while increased infiltration of Treg cells. In the low-risk group, the high mutation rates of DNAH5, FAT3, and FBXW7 genes resulted in increased infiltration of central CD4 + T lymphocytes and CD8 + T lymphocytes, while decreased infiltration of Treg cells, as shown in Supplementary Figure S1 and Supplementary Figure S2. We then further explored whether the high - and low-risk groups were associated with TMB, and our data demonstrated that the TMB was slightly higher in the low-risk group than that in the high-risk group (Figure 6C, *p* = 0.059).

### Immune Characteristics of Different Subgroups

**FIGURE 7:**
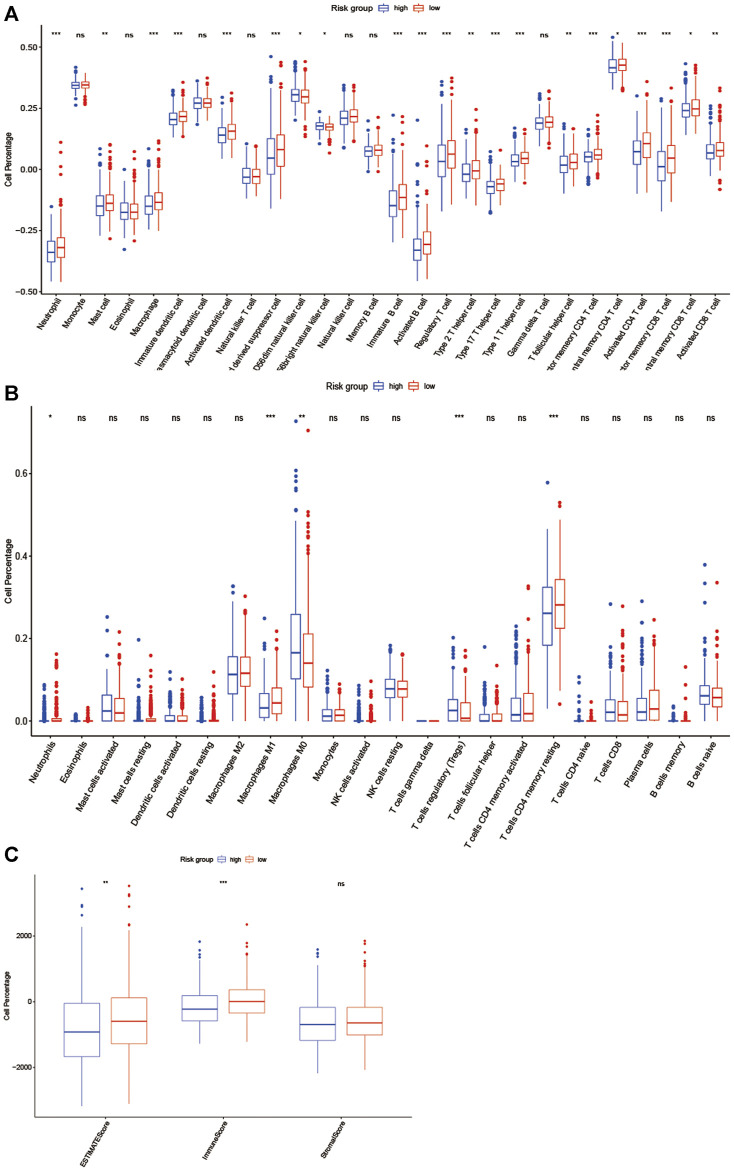
EMT-related lncRNA clusters significantly associated with the immune microenvironment. **(A, B)** Statistical differences in each type of immune cell between high-risk group and low-risk group using ssGSEA approach **(A)** and CIBERSORT algorithm **(B)**. **(C)**Stromal score and immune score were calculated via ESTIMATE method between high-risk group and low-risk group. (ns: not significant, *p < 0.05, **p < 0.01, ***p < 0.001).

We further evaluated the status of immune cell infiltration in TCGA colorectal cancer transcriptome using the ssGSEA approach, and 28 immune-related terms were incorporated to assess the abundance of immune cells in the tumor immune microenvironment. The results showed that 21 immune cell types were significantly different between the two groups (Figure 7A). Next, the CIBERSORT algorithm was performed to investigate the immune infiltration in CRC tissues between the high-risk and low-risk group. The results revealed that Neutrophils cells, macrophages M1 cells, T cell CD4 memory resting cells were more abundant in the low-risk group while macrophage M0 cells and T cells regulatory cells were more abundant in the high-risk group (Figure 7B). Subsequently, the ESTIMATE algorithm was performed, and we found that estimatescore and immunescore were much higher in the low-risk group than in the high-risk group, while there was no significant difference in stromalscore between the two groups. Therefore, those results indicate that there were more immune components in TME in the low-risk group (Figure 7C).

### The Benefit of ICI Therapy in Two Different Subgroups

**FIGURE 8:**
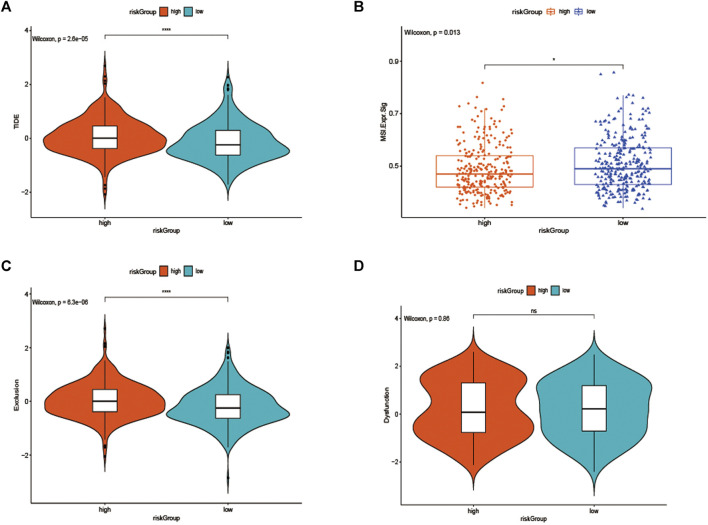
**(A)** TIDE, **(B)** MSI, and T cell exclusion **(C)** and dysfunction score **(D)** in the high-risk Group and low-risk group. The score between the two subgroups were compared through the Wilcoxon test (ns: not significant, *p < 0.05, **p < 0.01, ***p < 0.001).

As we knew, the higher TIDE prediction score represented a higher potential for immune evasion, which indicated that the patients were less sensitive to ICI therapy. Then TIDE was used to assess the potential clinical efficacy of immunotherapy in two groups. The results revealed that the low-risk group had a lower TIDE score than the high-risk group, indicating that the low-risk patients could benefit more from ICI therapy than those in the high-risk group (Figure 8A). Also, we found the low-risk group had a higher microsatellite instability (MSI) score (Figure 8B), while the high-risk group had a higher T cell exclusion score (Figure 8C), but there was no difference in T cell dysfunction between the two subgroups (Figure 8D).

## Discussion

Even with significant advances in screening and treatment strategies, CRC remains the second largest cause of cancer-related death around the world (Siegel et al., 2020). Therefore, a better understanding of CRC pathogenesis and exploring potential biomarkers will likely yield novel insights into the management and prognosis of CRC. In recent years, it has been widely revealed that EMT is closely related to cancer progression and metastasis (Mittal, 2018; Aiello and Kang, 2019), among them, abnormal development of genome, including lncRNA and mRNA, is a typical feature of regulating the tumor EMT process, in the present study, we aim to address the prognostic value of EMT-related lncRNAs in CRC.

Based on the TCGA dataset, we established an innovative and efficient EMT-related lncRNA signature, and then its validity was verified on the validation set, the ROC analysis results revealed its high prognostic value in two data set, besides, the signature showed a significant correlation with the TNM stage, furthermore, our data showed that low-risk group patients had a better OS. As the EMT-related lncRNAs were considered as a potential predictor for OS, as well as age and stage, a nomogram was constructed based on the above factors and showed promising performance in the 1-, three- and 5 years, which may distinguish individualized treatment of in CRC patients. In brief, our data revealed that a marvelous prognostic value of our EMT-related lncRNA signature, which may provide a theoretical basis for EMT-related targeted therapies for CRC. Moreover, in GSEA analysis, the results indicated that different pathways related to the progression of tumor were significantly enriched in the low-risk group and high-risk group, however, immune response-related pathways mainly enriched in the low-risk group and tumor metastasis-related pathways mainly plays a regulatory role in the high-risk group.

The EMT-related lncRNA signature was made up of 11 lncRNAs, and the molecular mechanism or prognostic value of them has not been exposed, due to their high prognostic value, subsequent experiments are needed to clarify their role in CRC.

Additionally, to explore biological characteristics of the subgroups in the training cohort, we then studied gene mutations of the high- and low-risk group. The results showed that missense variations were the most common type in the two groups, and significant variation differences between the two groups were APC and TP53, which were more common in the high-risk group than low-risk group (81 vs 73% and 64 vs55%), In Michael J et al. study, they revealed that APC and TP53 mutation is the most strongly negatively associated with MSI but positively associated with distant metastasis, which suggested a worse prognosis (Schell et al., 2016). Furthermore, in TIMER 2.0 database, we found that mutations in APC and TP53 genes can reduce the infiltration of CD4 + T lymphocytes and CD8 + T lymphocytes. In addition, we also found that high mutations in USH2A and NEB genes in high-risk groups lead to decreased infiltration of CD4 + T lymphocytes and CD8 + T lymphocytes in tumor centers and increased infiltration of Treg cells, which may be a factor leading to the characteristics of active immune response and low invasive tumor phenotype in patients in the low risk group. In the low-risk group, the high mutation rate of DNAH5, FAT3 and FBXW7 genes also led to the increase of central CD4 + T lymphocyte and CD8 + T lymphocyte infiltration and the decrease of Treg cell infiltration. Those may partly explain that the higher mutation rates of APC, TP53, USH2A and NEB genes lead to a worse survival prognosis in the high-risk group. What’s more, it is well known that TMB has been shown to be a potential biomarker for predicting ICI treatment response in many tumor types (Goodman et al., 2017; Jardim et al., 2021), our results revealed that the TMB was slightly higher in the low-risk group than that in the high-risk group, we thought that may partly explain the low-risk group was more sensitive to immunotherapy.

To further understand the immune characteristics of the two groups. The ssGSEA method was used to further evaluate the immune-cell infiltration status of TCGA colorectal cancer transcriptome, and the results suggested that neutrophils, macrophage M1 cells, T cells, and CD4 memory resting cells were enriched in the low-risk group, while M0 cells and T cell regulatory cells were more common in the high-risk group, numerous studies have shown that dense infiltration of T cells, especially cytotoxic CD8 T cells, and high density of M1 macrophages may be associated with acute inflammation, suggesting a good prognosis (Fuchs et al., 2019; Marcelis et al., 2020). In contrast, in many malignancies, M2 macrophages (the major subtype of macrophages) are associated with chronic inflammation and contribute to tumor growth and the development of aggressive phenotypes and have been associated with adverse outcomes (Mantovani et al., 2002; Yamaguchi et al., 2016), and it is noteworthy that our findings support these conclusions. Furthermore, according to the ESTIMATE algorithm, we identified that estimatescore and immunescore were much higher in the low-risk group, which suggests that the low-risk group had more immune components in TME, implying a favorable immunotherapy strategy.

It has been reported that TIDE is used to identify the underlying factors of two mechanisms of tumor immune escape: induction of T cell dysfunction in tumors with high cytotoxic T lymphocyte (CTL) invasion, and prevention of T cell invasion in tumors with low CTL levels (Wang et al., 2020b; Fu et al., 2020; Tsukada et al., 2020), interestingly, in our study, we also discovered that the low-risk group not only had a higher MSI score and lower TIDE score, but also had a lower T cell exclusion score, when compared to the high-risk group, even if there was no difference in T cell dysfunction between the two subgroups, those results suggested that these low-risk group patients had lower levels of immune escape and more MSI, and the higher mutational burden makes the tumor immunogenic and sensitive to PD1 therapy (Lin et al., 2020).

In the current study, we employed Univariate Cox analysis and LASSO algorithms to select significant candidate EMT-related lncRNAs for further multivariate Cox regression to construct the prognostic signature, and stratified analysis revealed that the signature was significantly associated with TNM stages. Furthermore, we used ssGSEA, CIBERSORT algorithm and the ESTIMATE method to assess the relative immune cell infiltrations of each sample. Differentially infiltration of immune cells and diverse tumor mutation burden (TMB) scores might give rise to the efficacy of lncRNA signature for predicting the sensitivity of immunotherapy for CRC patients. The effective signature we constructed was due to the TCGA database with sufficient tumor samples and complete clinical data.

Previous methods to study tumor immune microenvironment include immunohistochemistry and flow cytometry, both of which are inevitably limited to narrow views when comprehensively analyzing the composition of immune cells, and flow cytometry may lead to cytolysis of some cell types. In this study, the gene expression profile and clinical information of colorectal cancer were downloaded from TCGA database, and CIBERPORT, ESTIMATE and ssGSEA algorithm, general gene expression based evolutionary algorithm, are used to quantify cell components from gene expression profiles of large tissues. Therefore, different types of infiltrating immune cells can be quantified at the same time, so that the method avoids the concerns of various surface markers and possible cell separation. Of course, there are some limitations in using public database analysis, for example, in our study, we used the TIDE score to evaluate the potential clinical efficacy of the signature on immunotherapy, our results suggest that the TIDE score of the high-risk group is slightly higher than that of the low-risk group, but there is no significant statistical difference, this may be due to the insufficient number of CRC cases in TCGA database. Moreover, the signature also lacks external clinical samples to verify its effectiveness, which is also the disadvantage of using our method in public database, which depends on further improvement in future work. In one word, although the EMT-related lncRNA signature we developed is somewhat innovative, there are some limitations: the risk signature is established based on the TCGA public database, however, there is no strong external data to verify the effectiveness and practicability. Furthermore, the TCGA database was of limited size, and important clinical information was missing, which can lead to potential basis or errors.

## Conclusion

Collectively, our study developed and validated an EMT-related lncRNA signature that could be used as a certain reliable tool for predicting individual prognosis and decision-making in the treatment of patients with CRC.

## Data Availability

The original contributions presented in the study are included in the article/Supplementary Material, further inquiries can be directed to the corresponding author.

## Supplementary Material

Supplementary Figure 1
**(A–C)** The high mutation rate of USH2A, NEB genes in the high-risk group related to the decreased infiltration of CD4 + and CD8 + T lymphocytes, while increased infiltration of Treg cells, except CSMD3.

Supplementary Figure 2
**(A–C)** The high mutation rate of DNAH5, FAT3, and FBXW7 genes in the low-risk group related to the increased infiltration of CD4 + and CD8 + T lymphocytes, while decreased infiltration of Treg cells.

Supplementary Figure 3
**(A, B)** The higher mutation rate of APC and TP53 genes in the high-risk group led to the decreased infiltration of CD4 + and CD8 + T lymphocytes.

## References

[B1] Aiello N. M. Kang Y. (2019). Context-dependent EMT Programs in Cancer Metastasis. J. Exp. Med. 216 (5), 1016–1026. 10.1084/jem.20181827 30975895 PMC6504222

[B2] Chaffer C. L. San Juan B. P. Lim E. Weinberg R. A. (2016). EMT, Cell Plasticity and Metastasis. Cancer Metastasis Rev. 35, 645–654. 10.1007/s10555-016-9648-7 27878502

[B3] Diepenbruck M. Christofori G. (2016). Epithelial-mesenchymal Transition (EMT) and Metastasis: Yes, No, Maybe? Curr. Opin. Cel Biol. 43, 7–13. 10.1016/j.ceb.2016.06.002 27371787

[B4] Dongre A. Weinberg R. A. (2019). New Insights into the Mechanisms of Epithelial-Mesenchymal Transition and Implications for Cancer. Nat. Rev. Mol. Cel Biol 20 (2), 69–84. 10.1038/s41580-018-0080-4 30459476

[B5] Fabrizio D. A. George Jr T. J. Dunne R. F. Frampton G. Sun J. Gowen K. (2018). Beyond Microsatellite Testing: Assessment of Tumor Mutational burden Identifies Subsets of Colorectal Cancer Who May Respond to Immune Checkpoint Inhibition. J. Gastrointest. Oncol. 9, 610–617. 10.21037/jgo.2018.05.06 30151257 PMC6087857

[B6] Fu J. Li K. Zhang W. Wan C. Zhang J. Jiang P. (2020). Large-scale Public Data Reuse to Model Immunotherapy Response and Resistance. Genome Med. 12, 21. 10.1186/s13073-020-0721-z 32102694 PMC7045518

[B7] Fuchs Y. F. Sharma V. Eugster A. Kraus G. Morgenstern R. Dahl A. (2019). Gene Expression-Based Identification of Antigen-Responsive CD8+ T Cells on a Single-Cell Level. Front. Immunol. 10, 2568. 10.3389/fimmu.2019.02568 31781096 PMC6851025

[B8] Ghiringhelli F. Fumet J. D. (2019). Is There a Place for Immunotherapy for Metastatic Microsatellite Stable Colorectal Cancer? Front. Immunol. 10, 1816. 10.3389/fimmu.2019.01816 31447840 PMC6691024

[B9] Goodman A. M. Kato S. Bazhenova L. Patel S. P. Frampton G. M. Miller V. (2017). Tumor Mutational Burden as an Independent Predictor of Response to Immunotherapy in Diverse Cancers. Mol. Cancer Ther. 16 (11), 2598–2608. 10.1158/1535-7163.mct-17-0386 28835386 PMC5670009

[B10] Hugo W. Zaretsky J. M. Sun L. Song C. Moreno B. H. Hu-Lieskovan S. (2016). Genomic and Transcriptomic Features of Response to AntiPD-1 Therapy in Metastatic Melanoma. Cell 165, 35–44. 10.1016/j.cell.2016.02.065 26997480 PMC4808437

[B11] Jardim D. L. Goodman A. de Melo Gagliato D. Kurzrock R. (2021). The Challenges of Tumor Mutational Burden as an Immunotherapy Biomarker. Cancer Cell 39, 154–173. 10.1016/j.ccell.2020.10.001 33125859 PMC7878292

[B12] Kong J. Sun W. Li C. Wan L. Wang S. Wu Y. (2016). Long Non-coding RNA LINC01133 Inhibits Epithelial-Mesenchymal Transition and Metastasis in Colorectal Cancer by Interacting with SRSF6. Cancer Lett. 380, 476–484. 10.1016/j.canlet.2016.07.015 27443606

[B13] Ledys F. Klopfenstein Q. Truntzer C. Arnould L. Vincent J. Bengrine L. (2018). RAS Status and Neoadjuvant Chemotherapy Impact CD8+ Cells and Tumor HLA Class I Expression in Liver Metastatic Colorectal Cancer. J. Immunotherapy Cancer 6, 123. 10.1186/s40425-018-0438-3 PMC6245855 30454021

[B14] Lin A. Zhang J. Luo P. (2020). Crosstalk between the MSI Status and Tumor Microenvironment in Colorectal Cancer. Front. Immunol. 11, 2039. 10.3389/fimmu.2020.02039 32903444 PMC7435056

[B15] Ma X. Bi E. Lu Y. Su P. Huang C. Liu L. (2019). Cholesterol Induces CD8+ T Cell Exhaustion in the Tumor Microenvironment. Cel Metab. 30 (1), 143–156. Epub 2019 Apr 25. 10.1016/j.cmet.2019.04.002 PMC7061417 31031094

[B16] Mantovani A. Sozzani S. Locati M. Allavena P. Sica A. (2002). Macrophage Polarization: Tumor-Associated Macrophages as a Paradigm for Polarized M2 Mononuclear Phagocytes. Trends Immunol. 23 (11), 549–555. 10.1016/s1471-4906(02)02302-5 12401408

[B17] Marcelis L. Antoranz A. Delsupehe A.-M. Biesemans P. Ferreiro J. F. Debackere K. (2020). In-depth Characterization of the Tumor Microenvironment in central Nervous System Lymphoma Reveals Implications for Immune-Checkpoint Therapy. Cancer Immunol. Immunother. 69 (9), 1751–1766. 10.1007/s00262-020-02575-y 32335702 PMC11027603

[B18] Mittal V. (2018). Epithelial Mesenchymal Transition in Tumor Metastasis. Annu. Rev. Pathol. 13, 395–412. 10.1146/annurev-pathol-020117-043854 29414248

[B19] Nieto M. A. Huang R. Y.-J. Jackson R. A. Thiery J. P. (2016). Emt: 2016. Cell 166, 21–45. 10.1016/j.cell.2016.06.028 27368099

[B20] Schell M. J. Yang M. Teer J. K. Lo F. Y. Madan A. Coppola D. (2016). A Multigene Mutation Classification of 468 Colorectal Cancers Reveals a Prognostic Role for APC. Nat. Commun. 7, 11743. 10.1038/ncomms11743 27302369 PMC4912618

[B21] Siegel R. L. Miller K. D. Fedewa S. A. Ahnen D. J. Meester R. G. S. Barzi A. (2017). Colorectal Cancer Statistics, 2017. CA: A Cancer J. Clinicians 67 (3), 177–193. 10.3322/caac.21395 28248415

[B22] Siegel R. L. Miller K. D. Goding Sauer A. Fedewa S. A. Butterly L. F. Anderson J. C. (2020). Colorectal Cancer Statistics, 2020. CA A. Cancer J. Clin. 70 (3), 145–164. 10.3322/caac.21601 32133645

[B23] Terry S. Savagner P. Ortiz-Cuaran S. Mahjoubi L. Saintigny P. Thiery J.-P. (2017). New Insights into the Role of EMT in Tumor Immune Escape. Mol. Oncol. 11 (7), 824–846. 10.1002/1878-0261.12093 28614624 PMC5496499

[B24] Tsukada T. Kinoshita J. Oyama K. (2020). Dentification and Validation of Stromal-Tumor Microenvironment-Based Subtypes Tightly Associated with PD-1/pd-L1 Immunotherapy and Outcomes in Patients with Gastric Cancer. Cancer Cel Int 20, 92. 10.1186/s12935-020-01173-3 PMC7092673 32226313

[B25] Wang H. Yu M. Hu W. Chen X. Luo Y. Lin X. (2020). Linc00662 Promotes Tumorigenesis and Progression by Regulating miR-497-5p/AVL9 axis in Colorectal Cancer. Front. Genet. 10, 1385. 10.3389/fgene.2019.01385 32038723 PMC6993758

[B26] Wang Q. Li M. Yang M. Yang Y. Song F. Zhang W. (2020). Analysis of Immune-Related Signatures of Lung Adenocarcinoma Identified Two Distinct Subtypes: Implications for Immune Checkpoint Blockade Therapy. Aging (Albany NY) 12, 3312–3339. 10.18632/aging.102814 32091408 PMC7066911

[B27] Yamaguchi T. Fushida S. Yamamoto Y. Tsukada T. Kinoshita J. Oyama K. (2016). Tumor-associated Macrophages of the M2 Phenotype Contribute to Progression in Gastric Cancer with Peritoneal Dissemination. Gastric Cancer 19 (4), 1052–1065. 10.1007/s10120-015-0579-8 26621525 PMC5034006

[B28] Zhang L. Zhao Y. Dai Y. Cheng J.-N. Gong Z. Feng Y. (2018). Immune Landscape of Colorectal Cancer Tumor Microenvironment from Different Primary Tumor Location. Front. Immunol. 9, 1578. 10.3389/fimmu.2018.01578 30042763 PMC6048410

