# Supplementary figures and images for: Identification and Validation of EMT-Related lncRNA Prognostic Signature for Colorectal Cancer
Source: Front Genet. 2021 Sep 22;12:723802. doi: 10.3389/fgene.2021.723802 (PMC8513715; doi:10.3389/fgene.2021.723802)

A

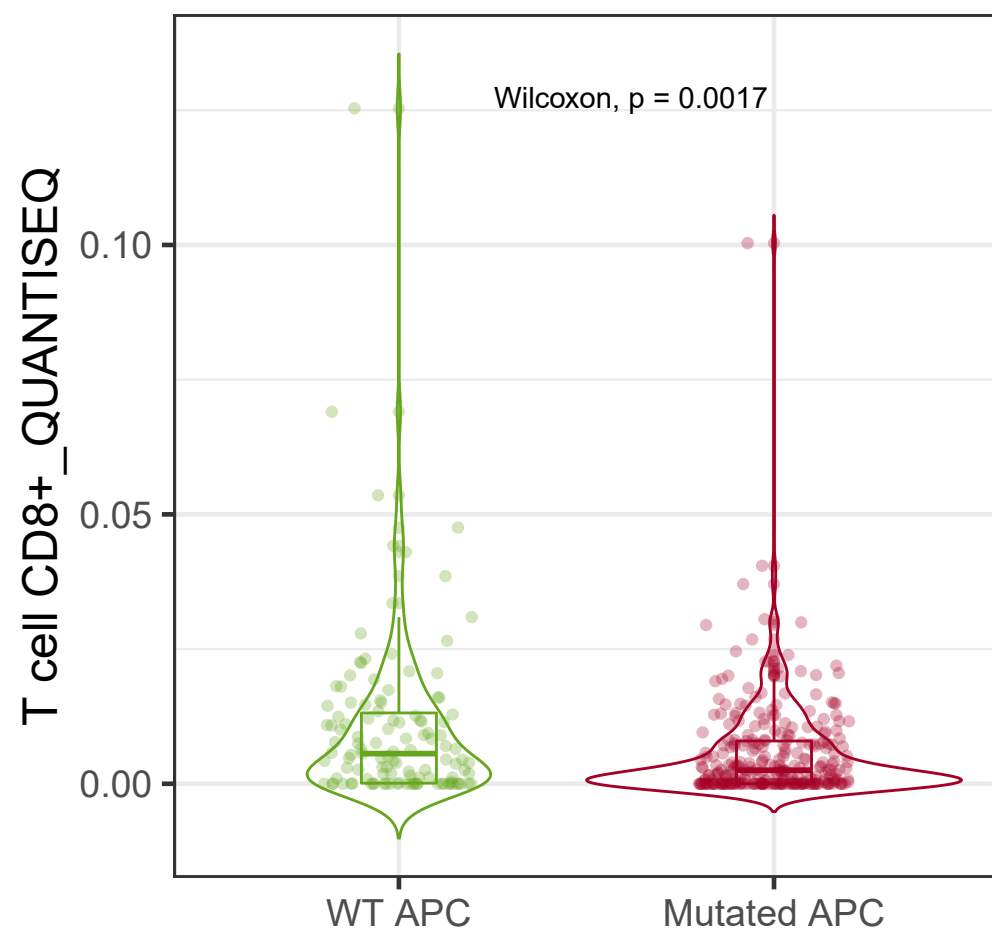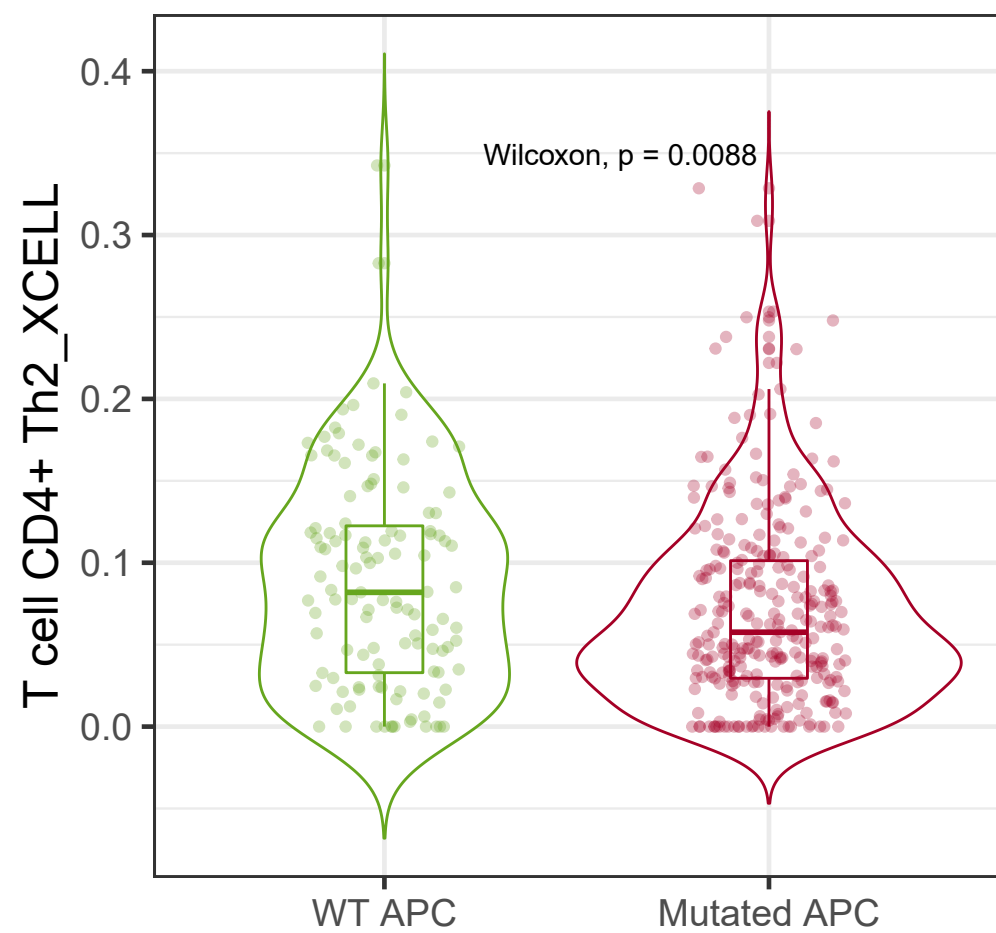

B

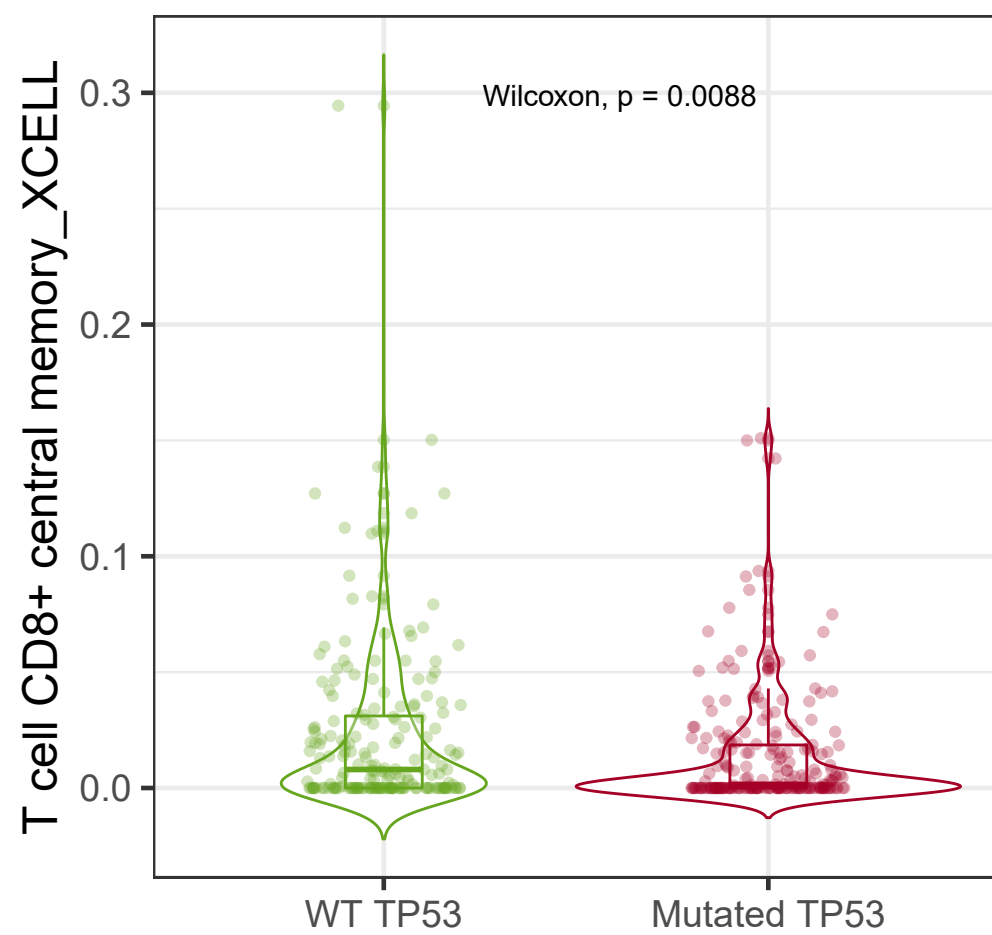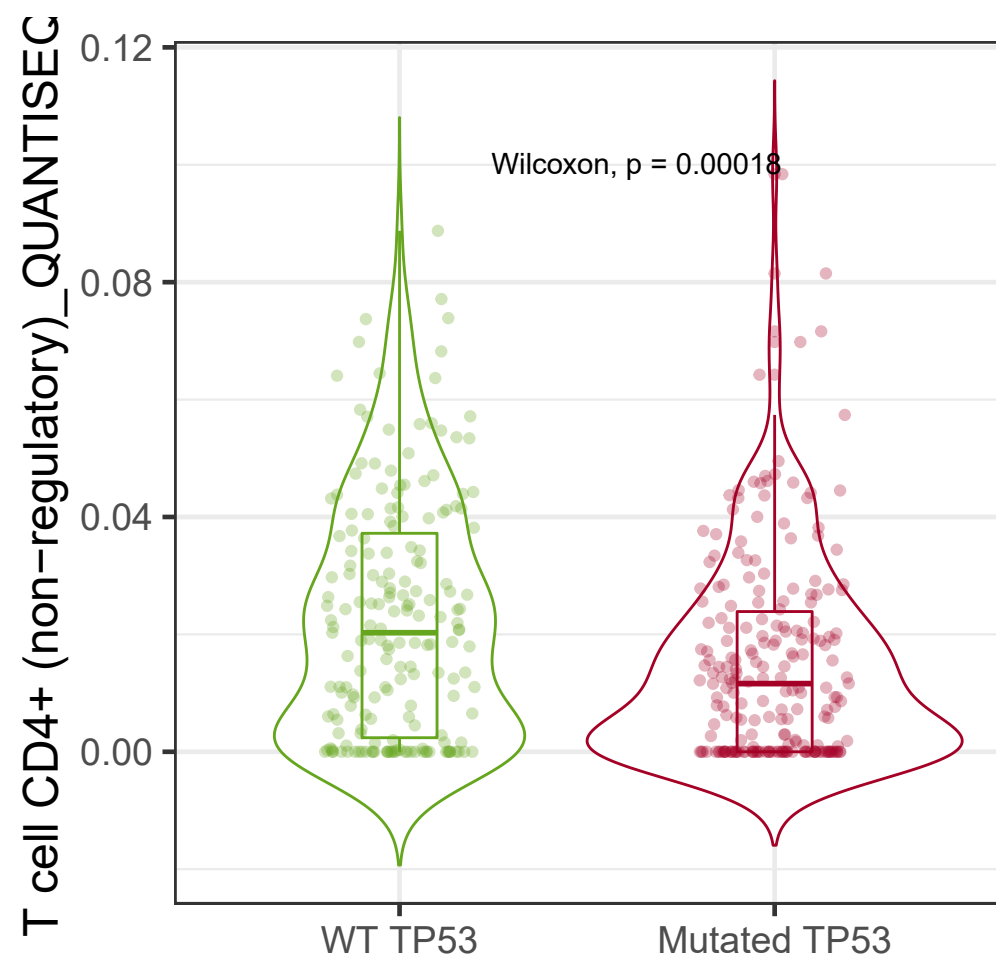

Supplement: Supplementary file 1 [file DataSheet2.PDF]

A

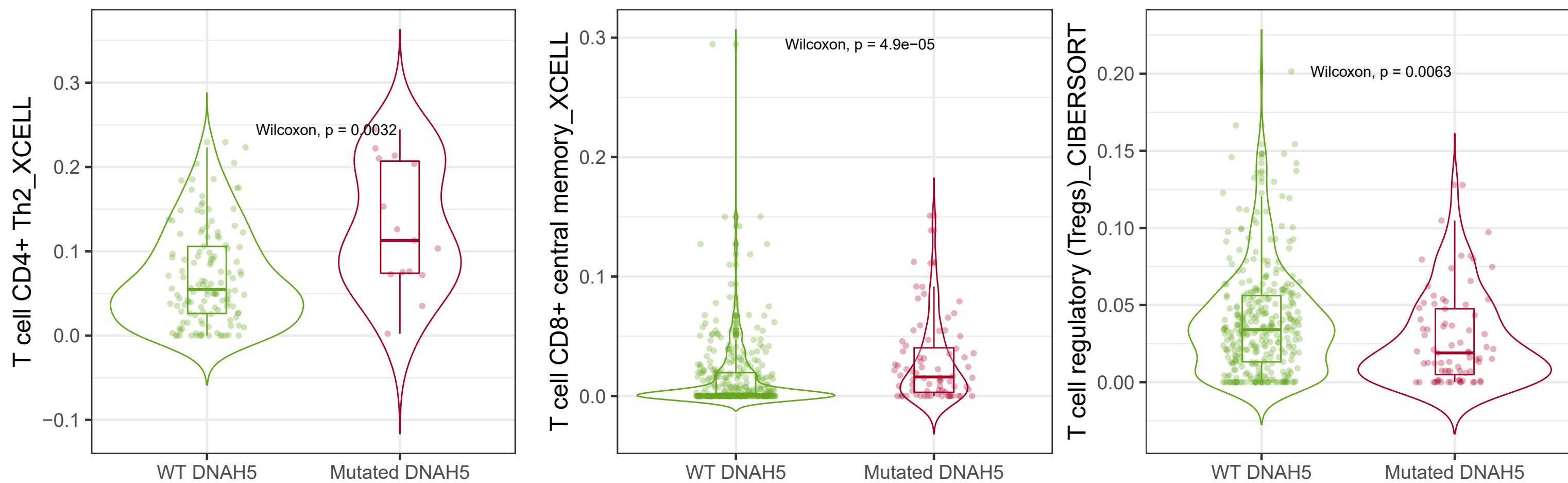

B

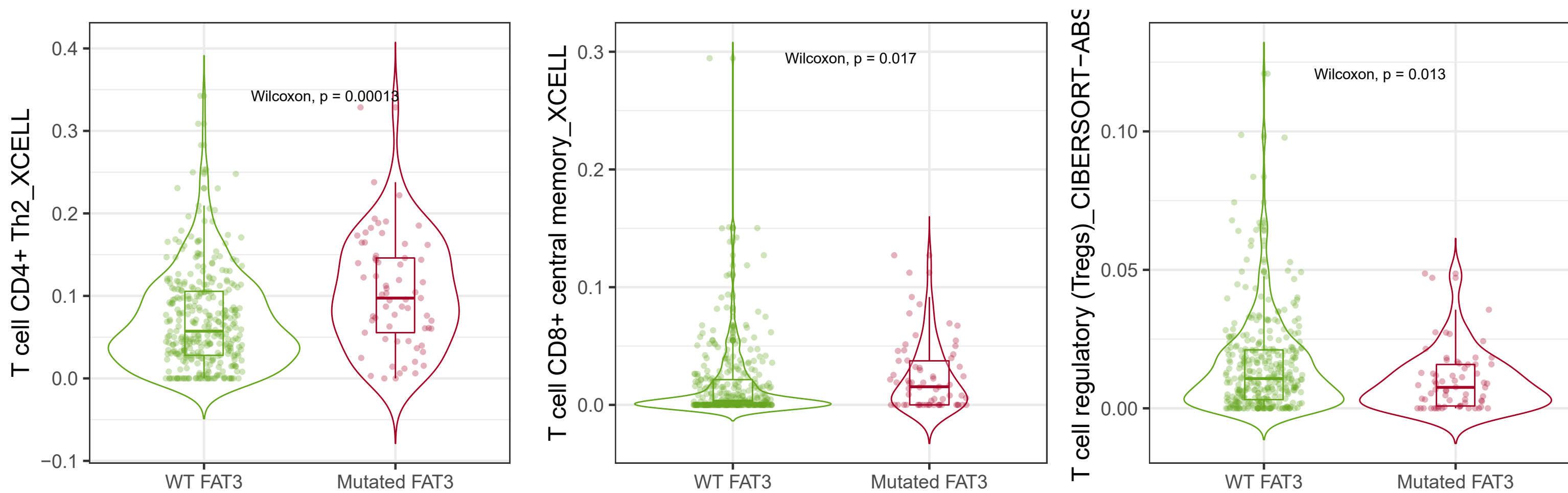

C

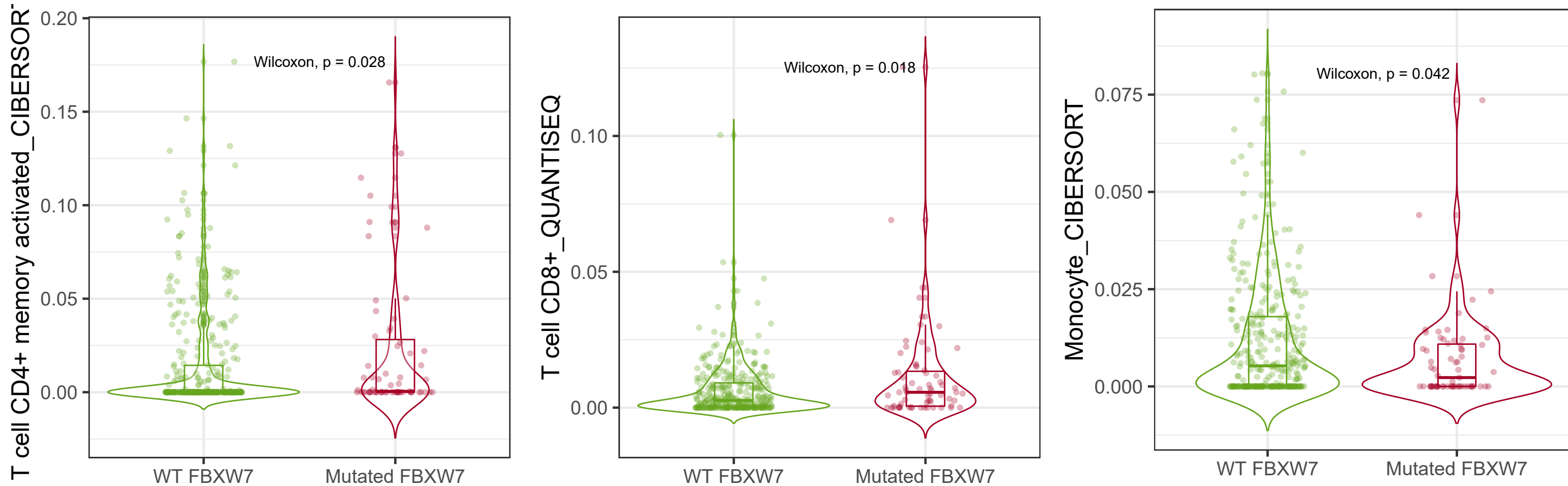

Supplement: Supplementary file 3 [file DataSheet3.PDF]

A

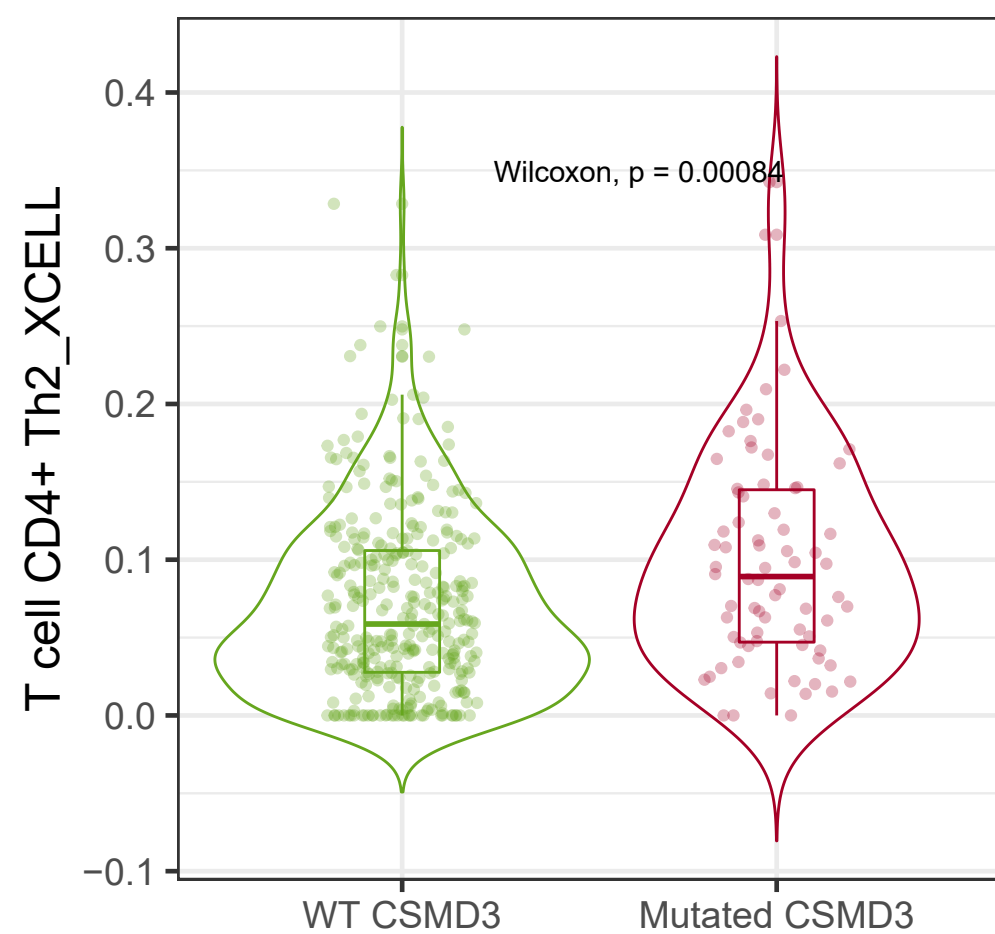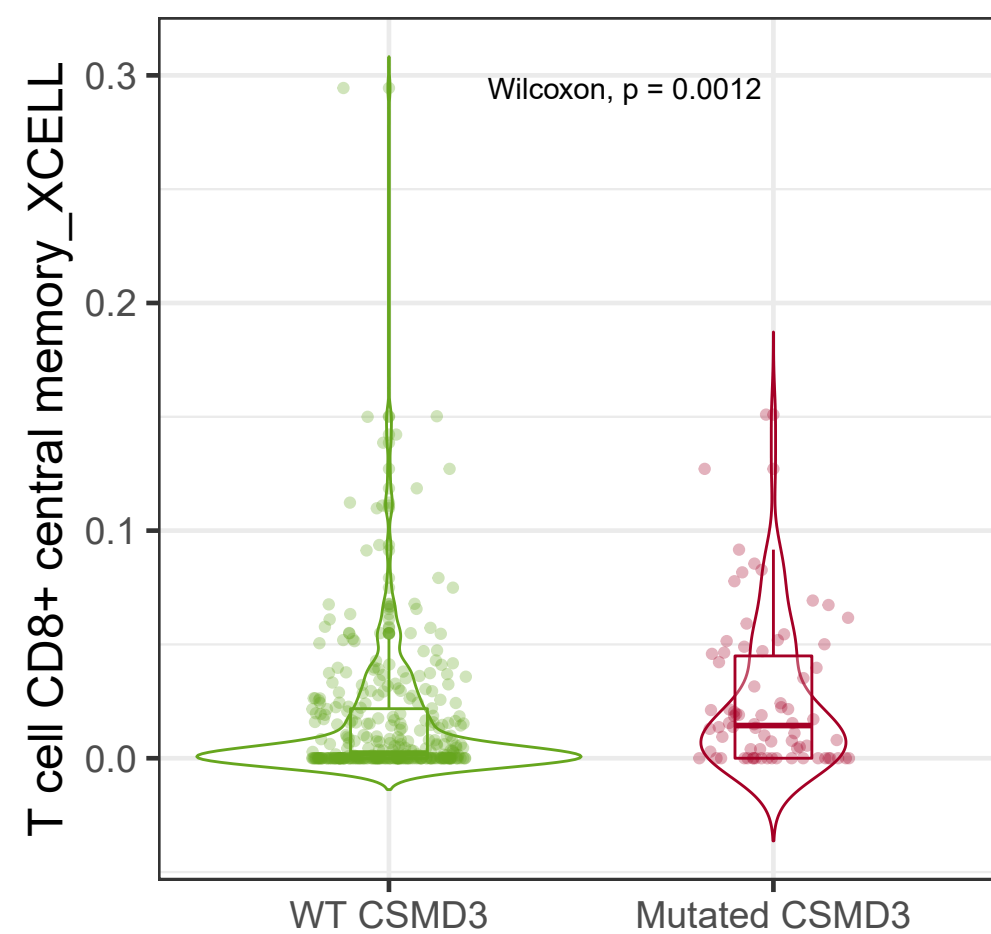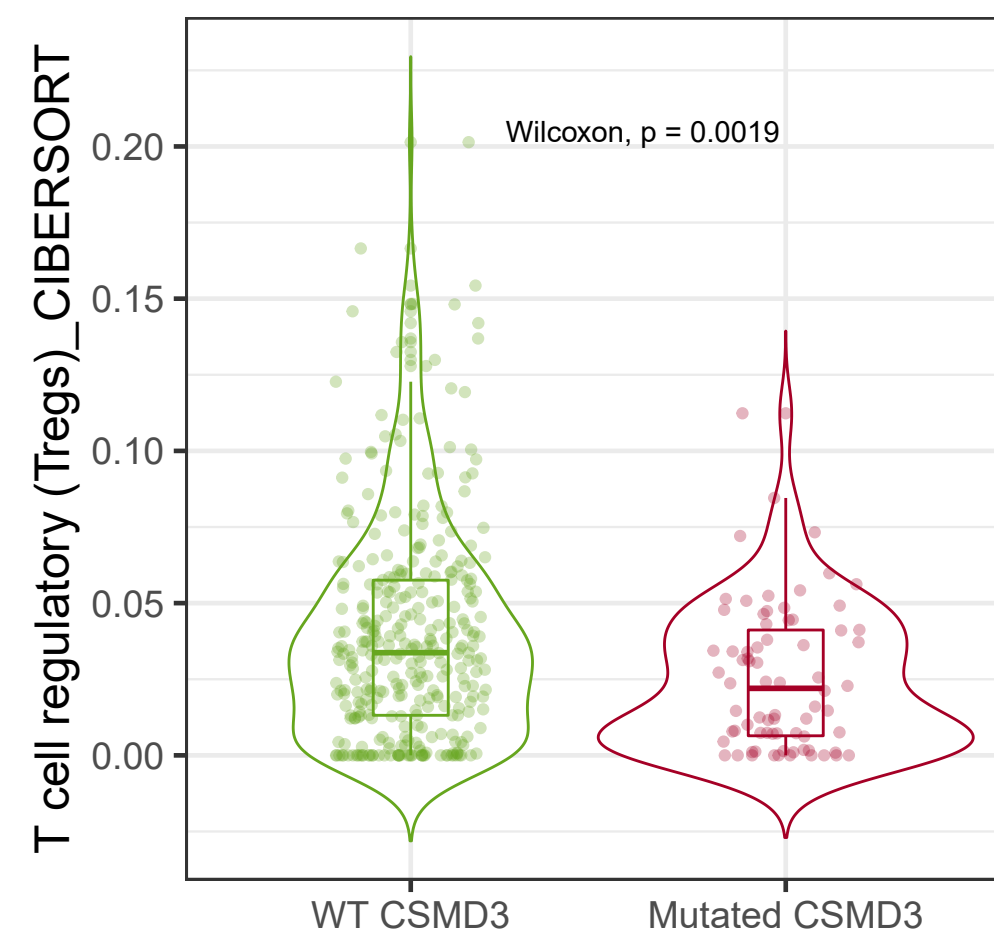

B

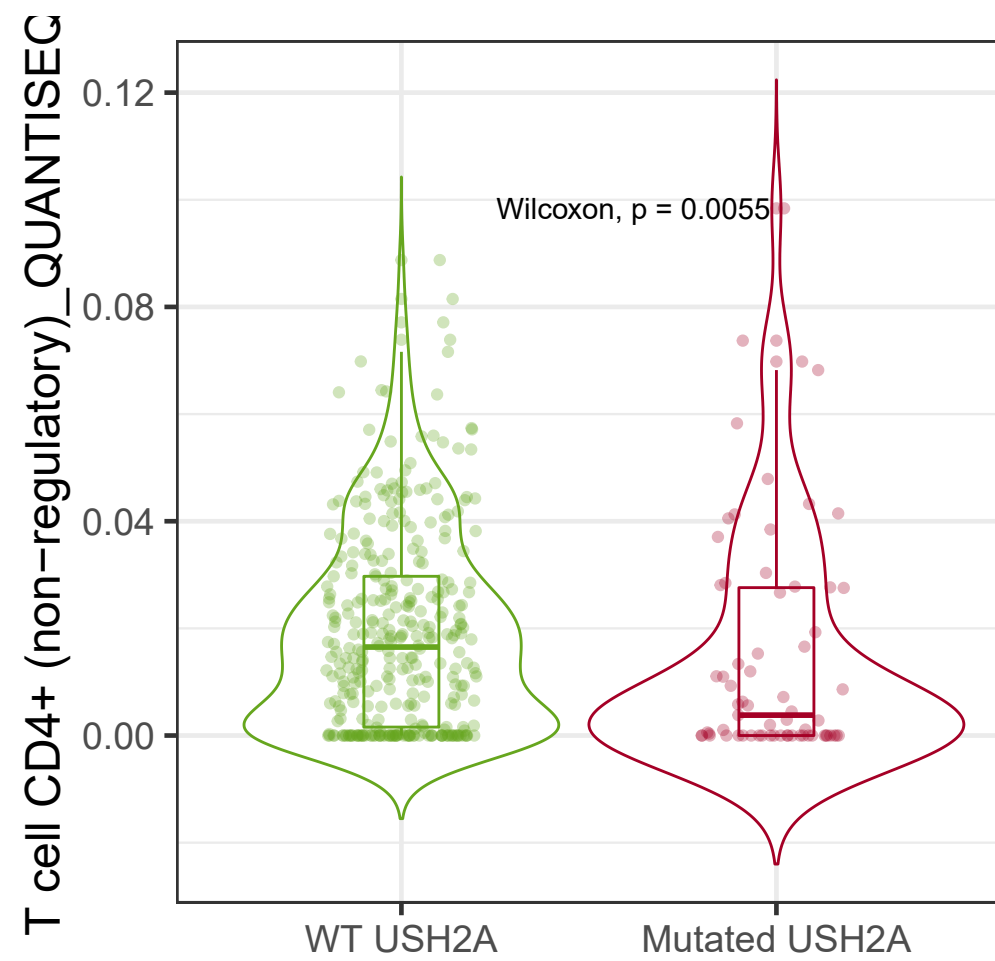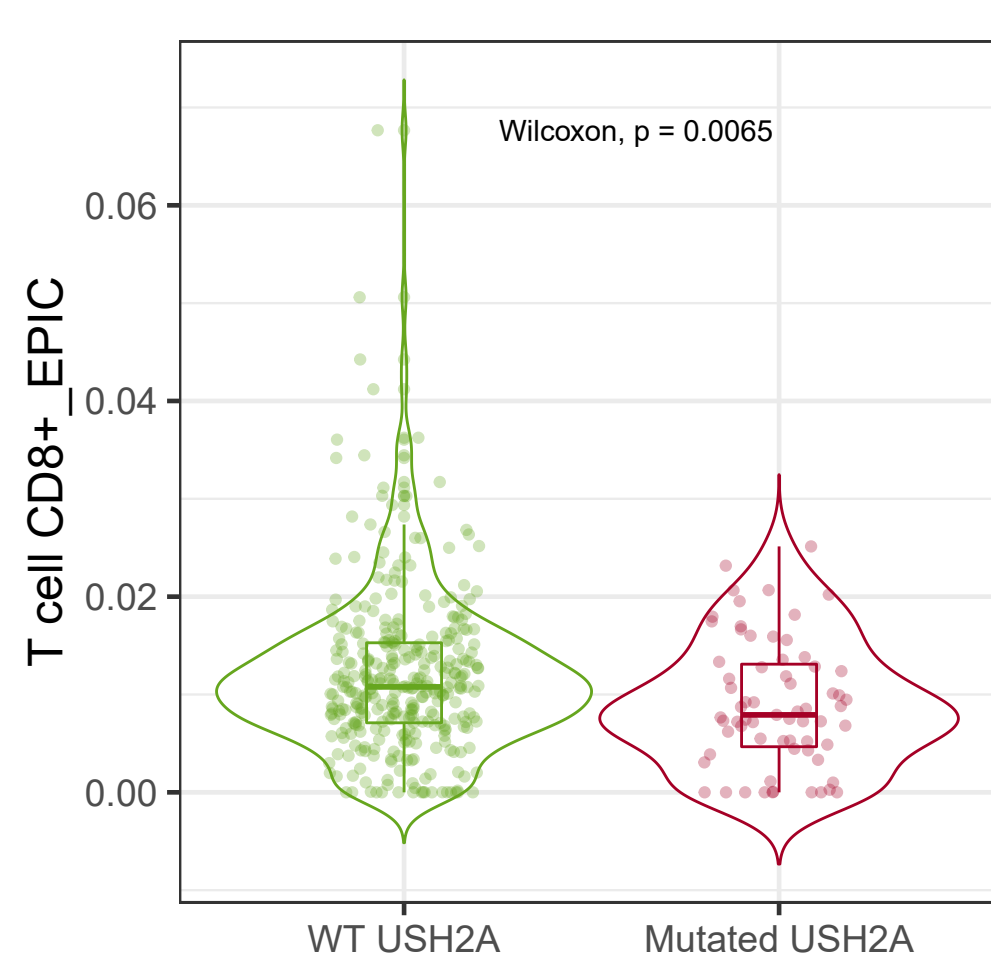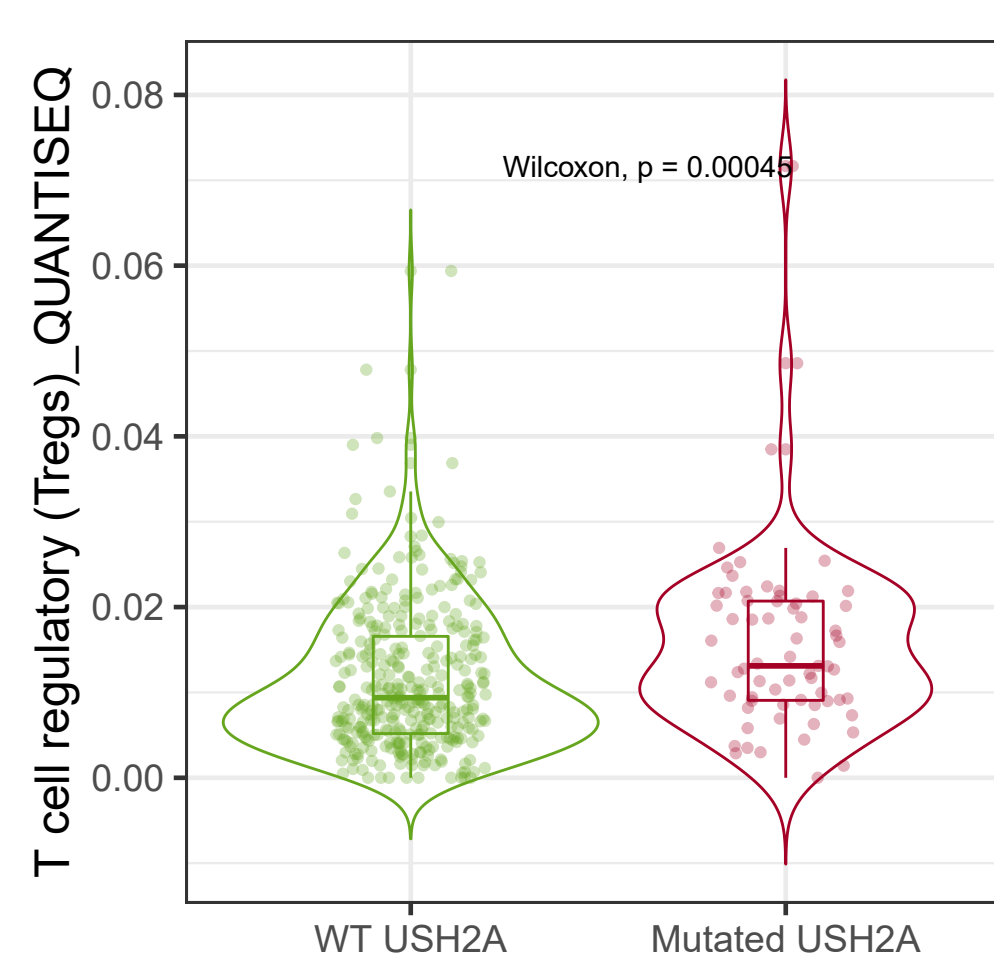

C

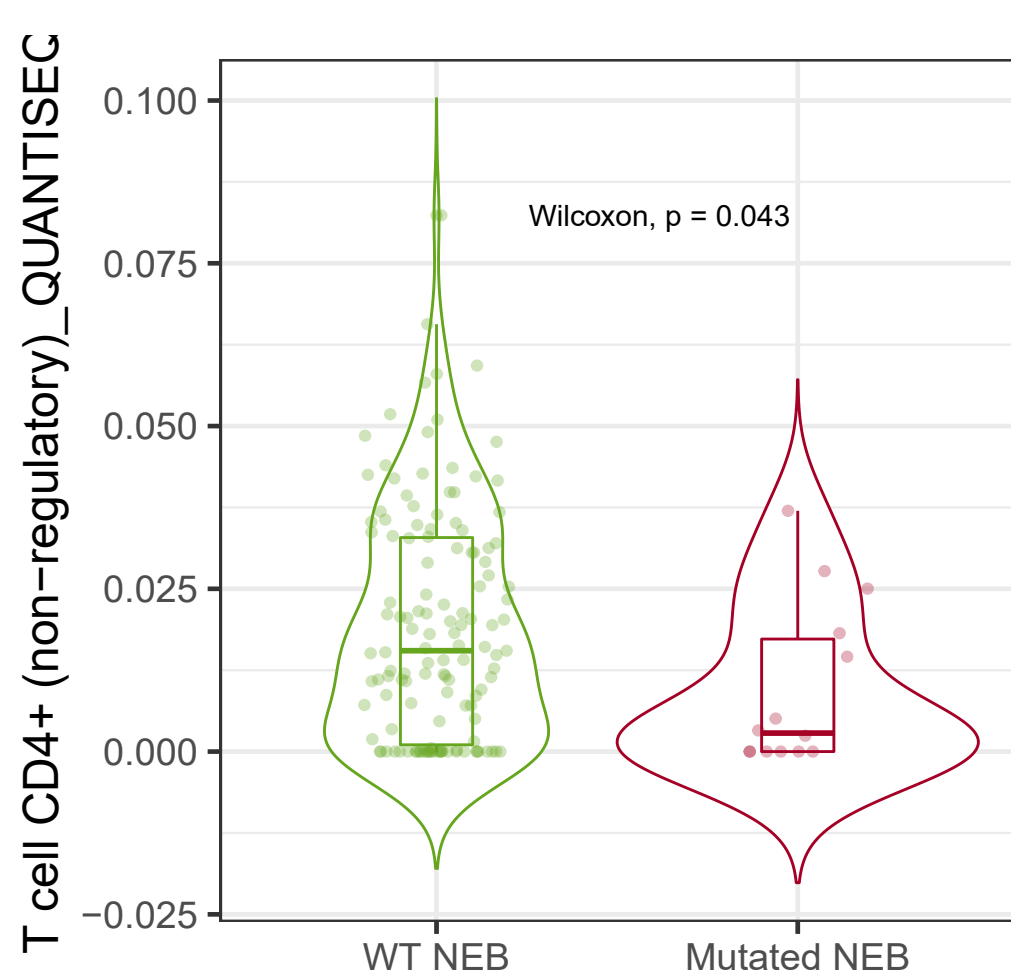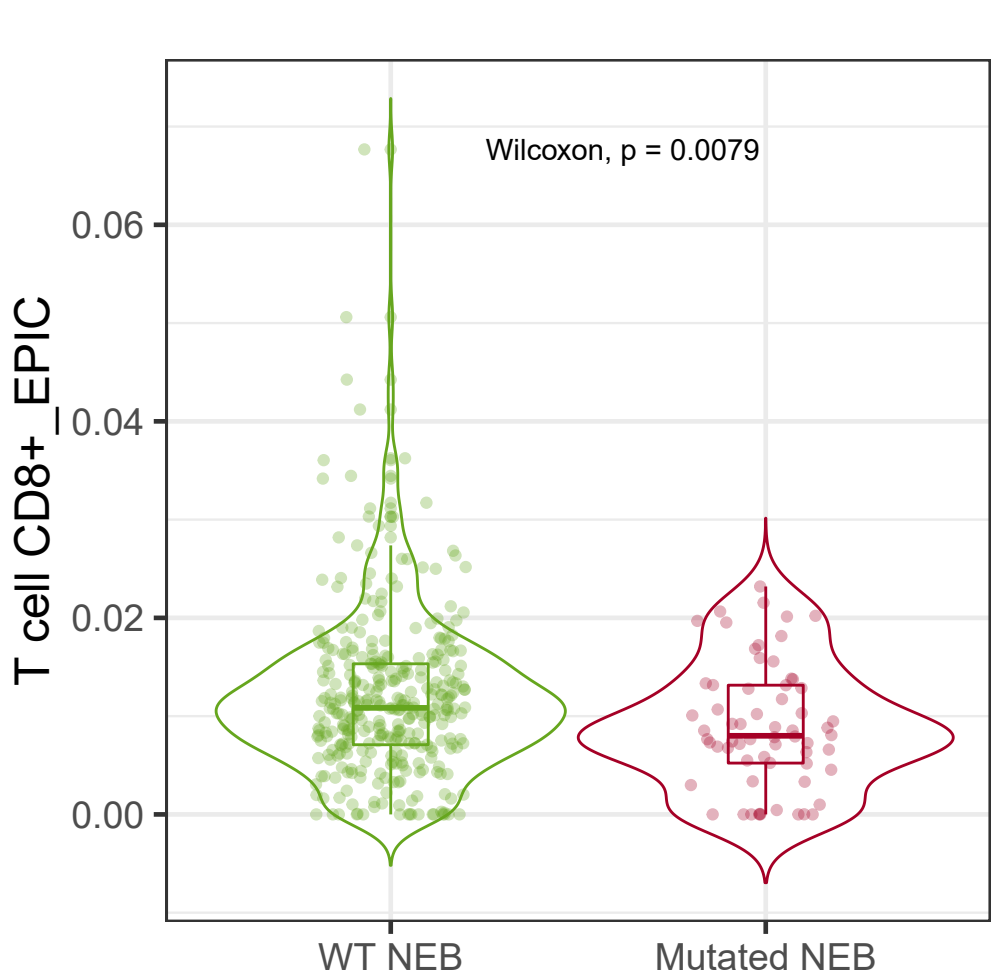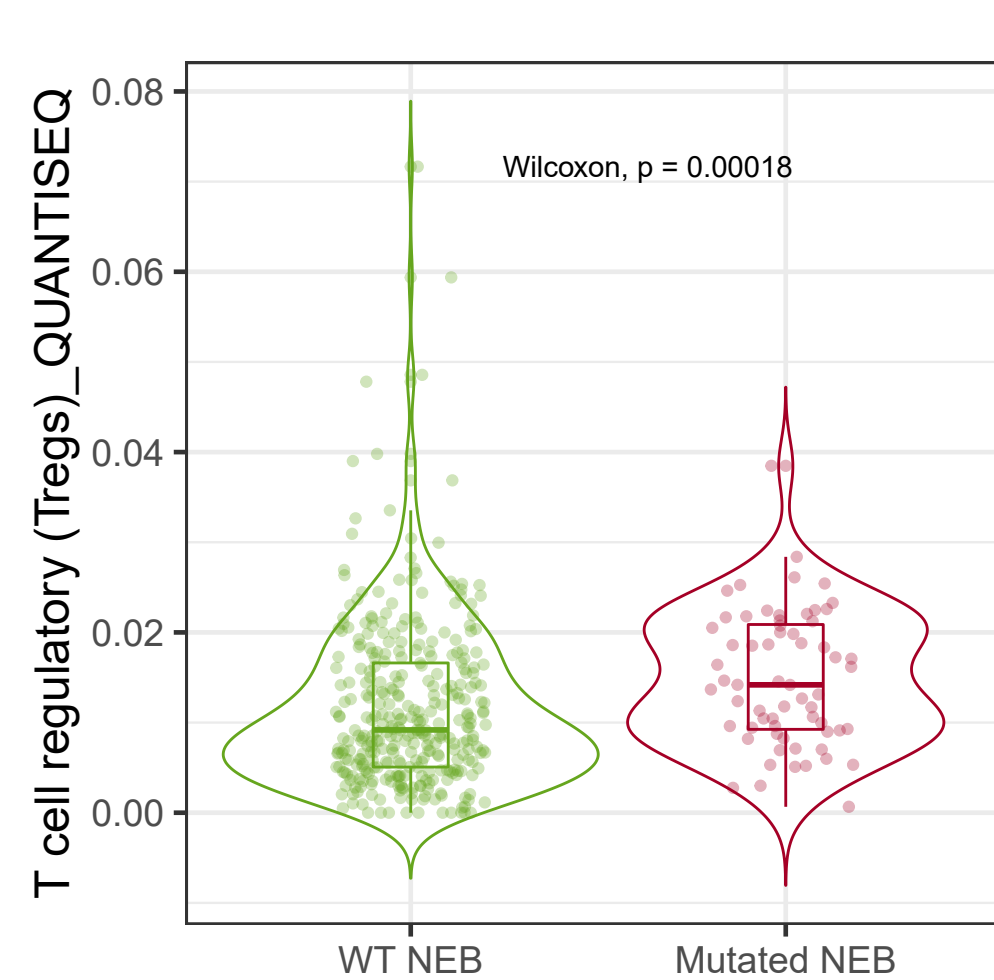

Supplement: Supplementary file 4 [file DataSheet1.PDF]
